# A framework for measuring timeliness in the outbreak response path: lessons learned from the Middle East respiratory syndrome (MERS) epidemic, September 2012 to January 2019

**DOI:** 10.2807/1560-7917.ES.2022.27.48.2101064

**Published:** 2022-12-01

**Authors:** Carolina dos S Ribeiro, Martine van Roode, Elmoubasher Farag, Mohamed Nour, Aya Moustafa, Minahil Ahmed, George Haringhuizen, Marion Koopmans, Linda van de Burgwal

**Affiliations:** 1 The Netherlands National Institute for Public Health and the Environment (RIVM), Center for Infectious Disease Control, Bilthoven, the Netherlands; 2 Vrije Universiteit (VU) Amsterdam, Faculty of Science, Athena Institute for Research on Innovation and Communication in Health and Life Sciences, Amsterdam, the Netherlands; 3 Erasmus Medical Center (EMC), Viroscience Department, Pandemic and Disaster Preparedness Centre, Rotterdam, the Netherlands; 4 Ministry of Public Health, Department of Public health, Doha, Qatar

**Keywords:** Infectious disease, outbreaks, zoonoses, data sharing, One Health, global health

## Abstract

**Background:**

Epidemics are a constant threat in the 21st century, particularly disease outbreaks following spillover of an animal virus to humans. Timeliness, a key metric in epidemic response, can be examined to identify critical steps and delays in public health action.

**Aim:**

To examine timeliness, we analysed the response to the Middle East respiratory syndrome (MERS) epidemic, with a focus on the international and One Health response efforts.

**Methods:**

We performed a historical review of the MERS epidemic between September 2012 and January 2019 in three steps: (i) the construction of a timeline identifying critical events in the global response, (ii) the performance of a critical path analysis to define outbreak milestones and (iii) a time gap analysis to measure timeliness in the execution of these milestones.

**Results:**

We proposed 14 MERS-specific milestones at different phases of the epidemic, assessing timeliness of the public health response as well as at the animal–human interface, where we identified the most significant delays.

**Conclusions:**

When comparing timeliness across three coronavirus epidemics, i.e. MERS (2012), SARS (2002) and COVID-19 (2019), we identified clear improvements over time for certain milestones including laboratory confirmation and diagnostics development, while this was not as apparent for others, as the identification of zoonotic hosts. To more efficiently respond to emerging threats, the global health community should widely assess and tackle specific delays in implementing response interventions by addressing challenges in the sharing of information, data and resources, as well as efficiency, quality, transparency and reliability of reporting events.

## Introduction

Infectious disease epidemics are occurring with increasing frequency and wider geographic range, on account of a combination of environmental, biological, socioeconomic and political drivers [1-5]. Mitigating the considerable socioeconomic impact of epidemics and disease outbreaks is dependent on effective and timely response to alerts triggered by the public health network and its partners. Essential for response is timely access to outbreak-related resources including diagnostic tools and other medical countermeasures, but most importantly biological material, data and remaining information. Significant delays in the production or sharing of knowledge related to new outbreaks translates into delays in implementing measures such as surveillance and the development of appropriate pharmaceutical and non-pharmaceutical countermeasures [6-8]. Such delays, however, are not uncommon. In the context of the coronavirus disease (COVID-19) pandemic, discussions were held on the timeliness of implementing response measures and how earlier implementation would have influenced the socioeconomic impact of the pandemic [9]. Similarly, evaluations of earlier outbreaks show that response is often suboptimal, and there is considerable room for improvement, especially in the aspect of timeliness [6-8,10-14].

An important factor contributing to suboptimal outbreak response is the complexity of actors, sectors and structures involved. For example, with 75% of all emerging infectious diseases constituted by zoonoses [1], effective communication and coordination across the public health, animal health and environmental sectors under a One Health (OH) approach is a key component of effective and timely responses [1,2,6,12,13,15]. Moreover, although national governments remain the primary actors and first line of defence for responding to epidemics, global coordination efforts that support timely epidemic management are also important (Box 1). For the actual collection and sharing of information and data, international expert centres and networks in different areas of infectious diseases management (Global Outbreak Alert and Response Network (GOARN), Pandemic Influenza Preparedness (PIP) Framework, R&D Blueprint strategy, Tripartite agreement) as well as open sharing platforms for genomic sequence data (International Nucleotide Sequence Database Collaboration (INSDC)), research data (bioarchives), and surveillance data (Programme for Monitoring Emerging Diseases (ProMED) have also been instrumental [2,6,7,10,11,13].

Box 1 **Examples of actors and initiatives for outbreak data collection and sharing**

**GOARN:** The Global Outbreak Alert and Response Network is a network under the World Health Organization (WHO) composed of technical and public health institutions, laboratories, non-governmental organisations (NGOs), and others that aims to surveil and respond to epidemics.

**PIP Framework:** The Pandemic Influenza Preparedness Framework is a network and instrument under the WHO that aims to enhance countries’ capacities to detect, prepare for and respond to pandemic influenza.

**R&D Blueprint:** The R&D Blueprint is a global strategy and network with stakeholders from medical, scientific and regulatory backgrounds under the WHO that allows the rapid activation of research and development activities during epidemics and fast-track the availability of countermeasures.

**Tripartite:** The Tripartite partnership for One Health brought together the Food and Agriculture Organization (FAO), the WHO and the World Organisation for Animal Health (WOAH) to develop and implement a joint plan of action to fight zoonotic and antimicrobial resistance threats. It has more recently become the Quadripartite with the inclusion of the United Nations Environmental Programme (UNEP).

**INSDC:** The International Nucleotide Sequence Database Collaboration consists of three main databases: the DNA Data Bank of Japan (DDBJ), National Center for Biotechnology Information’s GenBank (United States) and the European Nucleotide Archive ((ENA), United Kingdom), to collect and disseminate genomic sequence data through free and unrestricted access.

**Bio-archives:** Usually open access, preprint repositories for the biological sciences that publish papers before peer review but undergo basic screening and checks against plagiarism.

**ProMED:** The Programme for Monitoring Emerging Diseases of the International Society of Infectious Diseases (US) is one of the largest publicly available outbreak reporting system that provides up-to-date information concerning infectious disease outbreaks on a global scale.

The definition of ‘timeliness’, however, is still subject of debate, as outbreak response comprises a complex array of activities that are context dependent, do not always occur in a linear fashion and involve actors from different sectors [7,8,10,11,14]. In addition, ‘timely’ is not a fixed term and depends on the characteristics of the disease; the optimal turnaround times for surveillance data for instance depend on specific disease transmissibility, incubation period, duration of infectiousness and severity of outcomes as well as the objectives of the surveillance system in question [16]. In addition, the immediate impact of epidemics and outbreaks – socially, economically and/or geographically – directly influences the sense of urgency and the implementation of response measures [9]. Despite this complexity for standardisation, establishing a set of common indicators and metrics could greatly help to monitor and evaluate timeliness for outbreak response.

Timeliness can thus be defined based on the execution of different outbreak milestones, as applied by many studies [2,6-8,10-12,14,17]. This definition, however, depends on an analysis of timeframes for achieving outbreak milestones, which are scarce in current literature. In addition, although a number of studies have aimed to measure timeliness for outbreak response, all of them focus only on a few steps in the critical outbreak response path, with the majority relating to early surveillance actions.

The objective of this review is, therefore, to analyse timeliness in achieving outbreak milestones, focusing on an emerging zoonotic disease (EZD) epidemic of Middle East respiratory syndrome (MERS), which first arose in 2012. We examine the response throughout all epidemic phases [18], considering not only actions aimed at the public health response but also at the animal–human interface. In addition, we aim to identify how the flow of outbreak-related resources influences the timeliness in global epidemic investigation and response, with a focus on the international and OH MERS response efforts.

## Methods

We performed a historical review to analyse the timeliness of critical response steps in the MERS epidemic, which were defined by the achievement of outbreak milestones.

### Case study description

The MERS epidemic was chosen as a case for this study because its response relied heavily on collaboration across OH domains and international networks. MERS is an EZD caused by a coronavirus (MERS-CoV), likely originating from bats [19,20]. Although MERS-CoV infections have been identified in different animals such as sheep and cattle, dromedary camels have been demonstrated to be an important reservoir host and the main source of human infections [1,21]. There is no evidence of sustained MERS-CoV human-to-human transmission. However, MERS-CoV can be amplified in healthcare settings causing large nosocomial outbreaks [1], which have occurred in Saudi Arabia, United Arab Emirates, Qatar and South Korea [22]. Human MERS-CoV infections have been reported by 27 countries worldwide since 2012, with a total of 2,458 reported cases and 848 associated deaths [23]. The epidemic has never been declared a public health emergency of international concern (PHEIC) but is notifiable under the International Heath Regulations (IHR) of the World Health Organization (WHO) [1,24]. MERS-CoV infections in camels are also notifiable to the World Organisation for Animal Health (WOAH, previously known as OIE) under the World Animal Health Information System [25].

### Search strategy and selection criteria

In line with previous timeline studies [10,11], the primary search was based on the WHO public record of the Disease Outbreak News (DON), by screening the entire dataset for reports specific to MERS between September 2012 and January 2019, as well as the available archives from the official websites of the Food and Agriculture Organisation (FAO) and the WOAH. To complement the information available on these official websites, and to ensure that the earliest reporting date was recorded, snowball sampling was applied. The selected study sources (official reports) were used to identify and include further sources (additional literature). These additional literature sources consisted of grey and scientific literature such as peer-reviewed papers, meeting reports, published guidelines and press releases. Literature sources were included when they reported information related to the execution of at least one of the MERS milestones as predefined through the research frameworks and process and presented in the Results and Supplementary Material S1, indicating either when an event was first mentioned or complementing other sources by supporting the evidence. Although the date of reporting was generally specified in months, the date was indicated in days whenever possible. Literature sources were excluded when no information about the execution of milestones and critical response steps in the MERS response was reported, or if they were in a language other than English.

### Data analysis

The data analysis was performed in three steps: the development of an analytical timeline, the performance of a critical path analysis, and a time gap analysis. The construction of timelines is common practice to better understand outbreaks by identifying relevant events, their causes, development over time and their outcomes [26]. Nevertheless, there is no standardised way to define the types of applicable response measures and their order in the course and evolution of epidemics because of variation in pathogen-specific characteristics, affected populations, natural reservoirs and other outbreak-specific factors. For these reasons, we adapted two different frameworks for constructing and organising the timeline to the specific context and characteristics of MERS.

Firstly, the WHO framework of pandemic influenza defines four epidemic phases (i.e. alert, epidemic, transition, and interepidemic) based on the relative number of cases emerging [18]. To apply these phases for MERS and define their cut-off points, the MERS epidemic curve with the number of laboratory-confirmed cases reported to the WHO was used [27]. After adaptation of these frameworks for the MERS case study, five phases were defined as the following: (i) alert, (ii) I epidemic, (iii) II epidemic, (iv) transition, and (v) enzootic.

Secondly, the WHO framework for epidemic phases and response interventions was used to complement the description and definition of the different phases by applying four stages during the course of epidemics: introduction or emergence, localised transmission, amplification and reduced transmission [1]. This WHO framework was also used to chronologically categorise the types of response interventions in four steps: (i) anticipation, (ii) early detection, (iii) containment control and mitigation and (iv) elimination or eradication. These steps were used to thematically organise the critical events related to the MERS response in the timeline and later define the MERS milestones in the critical path analysis.

Within each of the five epidemic phases defined in the MERS timeline, the events were organised in a chronological fashion and clustered according to the type of response intervention to which they related, and whether they were targeted at the public health response (related exclusively to human health) or the response at the animal–human interface (including the animal health domain). To define milestones for MERS, critical steps on the execution of the epidemic response were selected from the timeline. Events were considered critical steps if they mirrored the inputs depicted in the WHO framework and the scientific literature [2,6-8,10,11,14,17] and, additionally, were found essential for the execution of a subsequent step in the response’s critical path.

Finally, to measure timeliness in the execution of the critical outbreak milestones, the earliest date on which the execution of the specific intervention/step was reported in the literature or communicated publicly was recorded and depicted in a bar chart. For instance, the emergence of MERS was defined by the detection of the first clinical case, although retrospective analysis showed that the disease emerged earlier but was diagnosed only later (see Supplementary Material S1. Description of the MERS analytical timeline for the phases: alert, I and II epidemic, transition, and enzootic and Supplementary Figure S1.1. Alert phase: MERS-CoV detection and primary response). A comparison of the timeliness of execution of the different outbreak milestones was then made between those targeted at the public health domain vs the animal–human interface.

## Results

### Data sources

The literature search resulted in 124 individual records, of which 60% (n = 75) were peer-reviewed literature and 40% (n = 50) were grey literature. From the grey literature, 33 were reports from the WHO website (of which 13 were DON, 11 were MERS-CoV updates and summaries, 3 were meeting reports and 6 were press releases and other documents), 6 were reports from the FAO website, 5 were reports from the WOAH website, 3 were media reports, and 3 were reports from other official government websites (Saudi Arabia, the United States and the European Union) and ProMED. The Supplementary Tables (S1.1 Descriptive list of the literature reviewed for the construction of the analytical timeline divided in types of literature; S1.2. Description of regional and international meetings that took place throughout the epidemic phases, and S1.3. Description of guidelines published by international organisations of WHO, FAO and WOAH throughout the epidemic phases) provide an overview of the included sources.

### The MERS timeline: defining MERS epidemic phases and critical response steps

**Figure 1:**
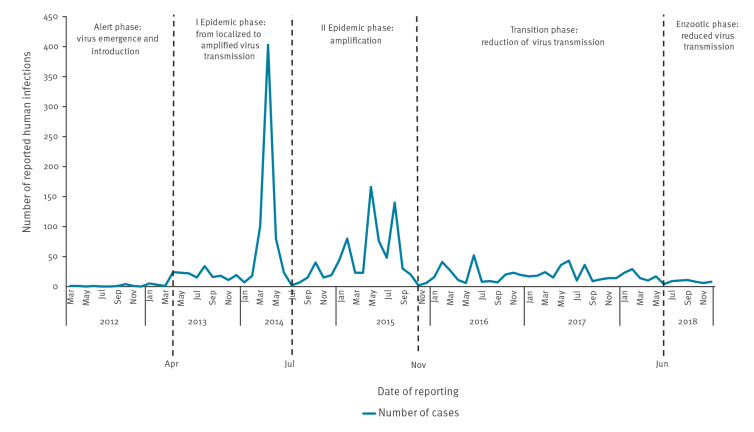
MERS epidemic phases based on the global number of laboratory-confirmed human infections per week according to the World Health Organization, May 2012–December 2018 (n = 2,266 infections)

Adapted from [1,18]. Number of laboratory cases was provided by the World Health Organization [27].

For the construction of the MERS timeline, we used the defined five adapted epidemic phases and the number of laboratory-confirmed cases [27] (Figure 1). A detailed description and visualisation of each of the five epidemic phases of the MERS timeline is provided in the Supplementary Material S1. Description of the MERS analytical timeline for the phases: alert, I and II epidemic, transition, and enzootic and Supplementary Figures S1.1. Alert phase: MERS-CoV detection and primary response, S1.2. I Epidemic phase: detection in camels and implementation of containment, control and mitigation strategies, S1.3. II Epidemic phase: containment, control and mitigation also at the animal-human interface, S1.4. Transition phase: the push for globally coordinated investigation, research and development, and S1.5. Enzootic phase: continued zoonotic transmission and emergence of cases.

#### Alert phase: MERS emergence and introduction of the virus

The first phase, defined as the alert phase, started in April 2012 and lasted until the end of March 2013. It was marked by the emergence of MERS-CoV and introduction into the human population through patient zero identified in Saudi Arabia. Although most cases were limited to the Arabian Peninsula, global concern was mounting due to the confirmation of human-to-human transmission and the exportation of cases outside the region. This phase is marked by the implementation of response interventions focused on early detection and initial containment of human infections such as laboratory confirmation, notification and reporting, case definition, diagnostics development, case finding and contact tracing, among others. For a more thorough presentation of the Alert phase, including details of patient zero and response measures implemented, see Supplementary Material S1 and Figure S1.1 (Alert phase: MERS-CoV detection and primary response).

#### I Epidemic phase: from localised to amplified MERS-CoV transmission

The I epidemic phase, from April 2013 until June 2014, was marked by an increase in the number of human cases with repeated outbreaks in the Arabia Peninsula region. This phase was characterised by a sharp peak reflecting frequent MERS-CoV spillovers from camels to humans and further amplification of human-to-human transmission mostly in healthcare settings. Measures targeted at containment, control and mitigation (e.g. case finding and contact tracing, management of infections, infection prevention and control, among others) were implemented at this point in both response streams (human and animal), while early detection measures were still being implemented at the animal–human interface (see Supplementary Material S1 and Figure S1.2. I Epidemic phase: detection in camels and implementation of containment, control and mitigation strategies).

#### II Epidemic phase: globally amplified MERS-CoV transmission

The number of cases was greatly reduced by June 2014, when a second surge in cases occurred 6 weeks later in the Arabian Peninsula region with four substantive peaks, defining the second epidemic phase (from July 2014 until October 2015). Moreover, towards the end of this phase, a large nosocomial outbreak emerged outside the region, in South Korea [22]. Progress in response interventions related to containment, control and mitigation measures were achieved, but early detection measures, such as activation of notification and reporting systems and the establishment of a case definition, were still being implemented at the animal–human interface (see Supplementary Material S1 and Figure S1.3. II Epidemic phase: containment, control and mitigation also at the animal-human interface).

#### Transition phase: reducing MERS-CoV human-to-human transmission

The transition phase was defined by a decrease in the number and size of human MERS-CoV outbreaks inside and outside the Arabian Peninsula region, lasting from November 2015 until June 2018. At this point, progress was still being made regarding response interventions targeted at detection, containment, control and mitigation of the epidemic such as coordination of global research and development activities, development of animal surveillance and investigation systems, among others. For a more thorough presentation of the transition phase, including detail on response measures implemented, see Supplementary Material S1 and Figure S1.4 Transition phase: the push for globally coordinated investigation, research and development.

#### Enzootic phase: reduced human-to-human but continued MERS-CoV zoonotic transmission

Lastly, the enzootic phase was defined from June until December 2018 as the interepidemic period (between potential epidemics) when no significant human outbreaks have occurred. Progress in response interventions targeted at epidemic containment were achieved, but regular individual zoonotic spillover events continued to occur in the Arabian Peninsula region (see Supplementary Material S1 and Figure S1.5 *.* Enzootic phase: continued zoonotic transmission and emergence of cases).

### The critical path analysis: defining outbreak milestones for the MERS response path

In the detailed description of the MERS epidemic timeline outlined in the Supplementary Material (S1. Description of the MERS analytical timeline for the phases: alert, I and II epidemic, transition, and enzootic and Figures S1.1–1.5), critical response steps – steps that enabled next steps in outbreak response to occur – are described in the order predefined in the WHO framework of types of response intervention [1]. Each of these critical steps represent a milestone for the MERS epidemic, with depicted steps related to the response at (i) the public health domain, and (ii) the animal-human interface. The critical response steps are summarised in the Table together with their description and the date representing the milestone execution or communication for both response domains. In addition, as the MERS milestones are aligned in accordance with the WHO framework on types of response interventions, it was used as an archetype to allow a parallel comparison with outbreak milestones as identified in the scientific literature [2,6-8,10,11,14,17].

**Table:** Overview of critical response steps including the MERS milestones for response at public health as well as the animal–human interface, April 2012–December 2018

Response interventions [1]	Outbreak milestones	Definition of timelines for the MERS public health response path	Date of execution	Definition of timelines for the MERS animal–human response path	Date of execution	Milestones identified in literature sources
Anticipation	Forecasting	Predicting the emergence of an outbreak in humans	Undefined	Predicting the emergence of an outbreak with zoonotic potential in animals	Undefined	Predictive alert of a potential outbreak [14]
	Identification of epidemic drivers	Identification of determinants and drivers of human outbreaks to inform early warning systems	Undefined	Identification of determinants and drivers of zoonotic outbreaks to inform early warning systems	Undefined	Undefined
	Development of preparedness plans	Development of preparedness plans for human outbreaks based on previous knowledge and experience	Undefined	Development of preparedness plans for outbreaks in animals with zoonotic potential based on previous knowledge and experience	Undefined	Prevention (enhanced surveillance or other intervention) [14]
Early Detection	Detection of emergence and introduction	Time between the first detection of an unusual event and the identification of the first human infection: from hospitalisation of P0 until realisation of unknown causative agent	Aug 2012	Time between the first detection of an unusual event and the identification of the first intermediary-host: from hospitalisation of P0 until reporting of camels with antibodies against MERS-CoV	9 Aug 2013	Index case [6], onset date [6,17], symptom onset [7,11,14], death [14], patient visit to physician [7,11,14], hospitalisation [14], threshold date [6], detection date [6,10,17], health event- occurrence, verification [8,14], identification, investigation [8], date of outbreak start [11,14], discovery [11], recognition [2]
	Laboratory confirmation	Time until pathogen identification and characterisation in humans: pathogen discovery in P0	Sep 2012	Time until pathogen identification and characterisation in intermediary-host: reporting of PCR-positive camels	11 Nov 2013	Laboratory confirmation [6,10,11], laboratory diagnosis [7], laboratory report of pathogen linked to a case [14]
	Notification and reporting	Time until the first public communication and/or reporting to public health authorities of an unusual public health event: ProMED-mail and activation of WHO/IHR notification system	20–25 Sep 2012	Time until the first public communication and/or reporting to animal health authorities of an unusual animal health event: WOAH request for notification	15 Jul 2014	Notification to WHO [11,17], report date [6], healthcare worker notification [7], low and high-level reporting and assessment [2], health event reported to (local, regional, national and/or international) authorities [8,14], WHO’s report [10,11], public communication date [6,10,11,14], declaration of an epidemic; alert raised [11]
	Case definition	Time until the publication of a case definition for infections in humans: by the WHO	25 Sep 2012	Time until the publication of a case definition for infections in animals: advice by WOAH expert group	15 Jul 2014	Undefined
	Diagnostics development	Time until publication of the first diagnostic assay for case finding in humans: RT-PCR	27 Sep 2012	Time until publication of the first diagnostic assay for case finding in animals: validation of serology for use in camels	Oct 2013	Undefined
Containment Control and Mitigation	Case finding and contact tracing	Time until publication of the first guidance for surveillance of human infections: by the WHO	12 Nov 2012	Time until publication of the first guidance for surveillance of animal infections: the Muscat Declaration	20 May 2014	Undefined
	Confirmation of transmission	Time until official communication of confirmed human-to-human transmission: combined investigations in Europe and Middle East	13 Feb 2013	Time until official communication of confirmed zoonotic transmission: by the WHO (website) on the MERS-CoV update report	27 Mar 2014	Undefined
	Management of infections	Time until publication of the first guidance for management of human infections: by the WHO	13 Mar 2013	Time until publication of the first guidance for management of animal infections: in the Muscat Declaration	20 May 2014	Undefined
	Microbiological investigation and research	Time until first publication/ reporting of molecular investigations in humans: sharing the first genomic sequence data of MERS-CoV from P0	Sep 2012	Time until first publication/ reporting of molecular investigations in animals: study on bats showing viral similarity with MERS-CoV	Jan 2013	Undefined
	Epidemiological/clinical investigations and research	Time until first publication/reporting of clinical and/or epidemiological investigations in humans: investigations in P0	Nov 2012	Time until first publication /reporting of clinical and/or epidemiological investigations in animals: sero-epidemiological study in camels	Oct 2013	Undefined
	Environmental investigation and research	Time until first publication reporting of investigations on potential animal/ environmental source of MERS-CoV: in bats	Jan 2013	Time until first publication /reporting of investigations on potential animal/ environmental source of MERS-CoV: in bats	Jan 2013	Undefined
	Joint risk assessment	Time until the communication of the first internationally agreed risk assessment with experts from multiple countries: first international meeting on MERS	13 Jan 2013	Time until the communication of the first multi-sectorial (OH) risk assessment: first meeting engaging the Tripartite partnership for One Health	Dec 2013	After-action review (by organisations from the Tripartite partnership for One Health) [14]
	Infection prevention and control (IPC) measures	Time until publication of the first guidance on IPC measures to prevent transmission within healthcare settings: by the WHO	15 May 2013	Time until publication of the first guidance on IPC measures to prevent zoonotic transmission: hygiene measures in the WHO’s DON website	29 Nov 2013	Undefined
	Global coordination of multi-country studies	Time until the coordination of multinational efforts to support outbreak research and development: in the first Tripartite partnership for One Health meeting	15 Dec 2013	Time until the coordination of multinational efforts to support outbreak research and development: in the first Tripartite partnership for One Health meeting	15 Dec 2013	Undefined
Elimination or Eradication	Interrupt transmission	Interrupt human-to-human transmission	Not achieved	Interrupt zoonotic and animal-to-animal transmission	Not achieved	Date of end of the outbreak (declared by a responsible authority) [14,17];

DON: Disease Outbreak News; IHR: International Health Regulations; IPC: infection prevention and control; MERS: Middle East respiratory syndrome; MERS-CoV: MERS coronavirus; OH: One Health; ProMED: program for monitoring emerging diseases; P0: patient zero; Tripartite partnership for One Health is composed of: Food and Agriculture Organization (FAO), World Health Organization (WHO) and World Organisation for Animal Health (WOAH).

The milestones identified for the MERS case study are described according to the events used to estimate timeliness in their execution or communication, reported by date in columns 3 and 4 when related to the public health domain (aligned with the blue boxes in Supplementary Material S1 and Figures S1.1–5, including literature references), and in columns 5 and 6 when related to the animal–human interface (aligned with the green boxes in Supplementary Material S1 and Figures S1.1–5 including literature references). Outbreak milestones identified in the literature are also presented according to the types of response intervention they address [2,6-8,10,11,14,17].

The milestones grouped under the heading ‘Anticipation’, which include forecasting, identification of epidemic drivers and development of preparedness plans, have undefined execution dates because these are constant efforts that must be in place before, during and after epidemics, and involve steps taken in previous epidemics as well as steps that feed into future ones. For the MERS milestones related to early detection, containment, control and mitigation, all were executed within 2 years from the epidemic onset (from the alert phase until the second epidemic phase). The milestones that were exclusively related to the public health response were all executed within the alert and the first epidemic phases. In contrast, the milestones focused on the response at the animal–human interface were executed until the second epidemic phase, with most milestones being executed during the first epidemic phase. Milestones focused on eradication and elimination were not achieved for MERS because of the fact that pharmaceutical interventions that eliminate transmission, i.e. veterinary and human vaccines, are not yet available. Moreover, even if such vaccines become available, complete elimination would be very difficult as it would depend on high levels of efficacy and adoption of such interventions in both public health and animal health systems. Therefore, from the total of 18 outbreak milestones described in the Table, 14 were defined for the MERS epidemic, as the four milestones associated with response interventions of Anticipation and Elimination/Eradication could not be defined.

### The time gap analysis: measuring the timeliness in the execution of MERS outbreak milestones

**Figure 2:**
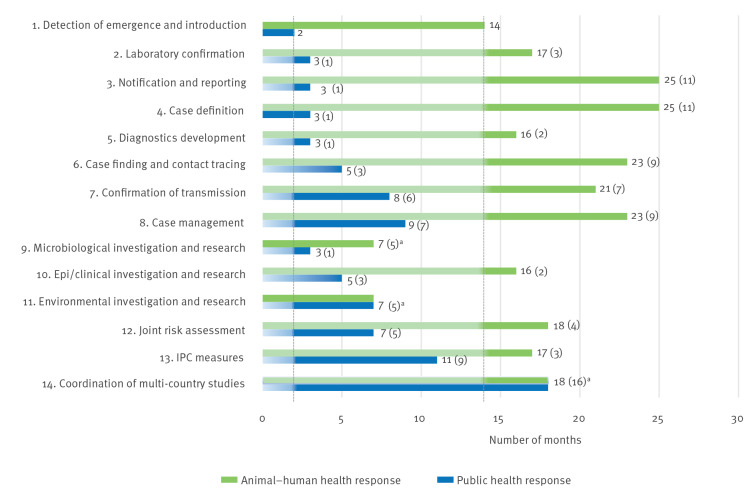
Time gap analysis measuring the timeliness for the execution of outbreak milestones of the MERS response, April 2012–December 2018 (n = 14 milestones)

IPC: infection prevention and control; MERS: Middle East respiratory syndrome.

^a^ Milestone at the animal–human interface that used the same reference as their correspondent at the public health domain because their execution is independent of the pathogen detection in animals.

In Figure 2, the time gaps for the execution of each of these MERS milestones are shown, allowing a comparison between the interventions targeted at the public health response versus the animal–human interface. The time intervals measure the absolute difference in time between the date of the epidemic onset (hospitalisation of patient zero) and the first date in which the execution of a critical step related to an outbreak milestone was reported, as well as the relative time gap in relation to pathogen detection in humans (month 2) and camels (month 14). Three milestones, microbiological investigation (Milestone 9), environmental investigation (Milestone 11) and coordination of multi-country studies (Milestone 14), used the same starting point for both response – the human and animal–human interface – because they were initiated with the pathogen discovery and suspicion of a zoonotic link, and thus were independent of the identification of MERS-CoV in camels.

The MERS milestones are numbered according to the detailed timeline outlined in the Supplementary Material S1 and Figures S1.1–5). The blue bars represent the milestones targeted at the public health response (exclusive to the human health domain), while the green bars represent the milestones targeted at the response at the animal–human interface (also including the animal health domain). The time intervals measure the absolute difference in time between the date of the epidemic onset (13 June 2012: hospitalisation of patient zero) and the first date in which the execution of a critical step related to an outbreak milestone was reported (numbers after the bars) in months. The relative time intervals (darker shadings and numbers in parentheses after bars) measure the difference in time between the execution of milestones and the timepoint of pathogen detection in humans (timepoint of 2 months, represented by left dashed line) and in the animal reservoir (timepoint of 14 months, represented by right dashed line).

With the epidemic onset as a reference point, most of the milestones at the animal–human interface took significantly longer to be executed than the ones focused on the public health response. However, when considering the time gaps relative to the pathogen detection in humans or camels, most milestones (Milestones 2–8) took more time to be executed in the animal health domain, while some milestones including epidemiological and clinical investigations (Milestone 10), joint risk assessment (Milestone 12), and infection prevention and control (IPC) measures (Milestone 13) took more time in the public health domain. The most evident delays, when comparing both responses, occurred at the animal–human interface for notification and reporting (Milestone 3), case definition (Milestone 4), followed by case finding and contact tracing (Milestone 6).

## Discussion

This review identified 14 MERS-specific milestones that signal timeliness for executing response steps in the public health domain, and at the animal–human interface. This comparison enables the identification of critical domain-specific delays related to the international coordination level of response. Only one other study [14] was identified in the literature that accounted for outbreak milestones considering the OH context, but those were still restricted to early surveillance actions. This historical review goes beyond existing approaches to describe milestones proceeding early surveillance – related to outbreak containment, control and mitigation – detailing critical steps in outbreak response. Assessing timeliness in the execution of these milestones can be used to compare outbreak response across domains as well as across epidemics, highlighting the most significant delays that need to be addressed for future efforts.

The current review identified new milestones that have not been previously described to assess timeliness yet are part of a critical path in outbreak response. Two of these new milestones, case definition and (host-specific) diagnostic development, occur in the phase of early detection and initial containment measures. These are considered critical steps because the development of a diagnostic assay and case definition allow for case finding and the activation of surveillance activities [28]. During outbreak containment, control and mitigation, proposed milestones are case finding and contact tracing, confirmation of transmission, management of infections and IPC measures. These are critical interventions that were implemented in the context of MERS and have their individual importance but are also connected in a critical path: case finding and contact tracing are key for understanding transmission, and confirming transmission routes is essential to develop actions for preventing and managing infections [1].

Research activities, either in the form of epidemiological, clinical, microbiological or environmental investigations, were also identified as milestones from the MERS response. Epidemiological and clinical studies were essential for answering questions and developing control measures considering factors like disease severity, transmission routes and risk factors [19,29-31]. Microbiological and environmental studies not only supported pathogen detection and identification, but also understanding of transmissibility, pathogenicity and efforts for the development of pharmaceutical countermeasures [20,29-39]. Finally, international and cross-sectorial coordination efforts, which are important for developing risk assessments and multinational studies, were identified as milestones. These interventions mark the engagement of relevant stakeholders from diverse sectors and regions on response efforts and the mobilisation of global expertise to support faster epidemic control [19,21,29,40-47].

When considering the epidemic onset as a reference, the execution of milestones at the animal–human interface faced substantially longer delays than those concerned with the public health response. Nevertheless, when based on pathogen emergence in specific camel and human populations, some milestones for the animal health response were executed faster than their respective milestone in the public health response. As an example, the milestone concerning implementation of IPC measures in the public health domain was the most delayed, comparatively. The timeliness of response in the animal health domain is strongly influenced by the disease profile. As MERS-CoV infection is not associated with any significant disease in dromedary camels, the urgency for response steps is not driven by sector-specific needs. There are, therefore, three crucial observations regarding timeliness in case of EZD outbreaks: (i) how fast the intermediary-host is identified, (ii) whether the pathogen constitutes an animal health threat and (iii) from that point on, the speed at which the necessary response measures were taken.

For MERS, although the suspicion and initial investigation of an animal reservoir took place early in the epidemic, the first evidence of camel infection was reported almost a year later [30]. This highlights the critical role of identifying intermediary animal populations of zoonotic relevance since, without this milestone, a set of other critical steps cannot be initiated. For some milestones, however, this delay persisted even after the detection of MERS-CoV in camels, as it took another 11 months for the official activation of the WOAH notification and reporting system for camel infections and the first guidance on a case definition [25]. The late recognition of camels as an intermediary host for MERS-CoV also influenced the development of guidance on case finding, contact tracing and case management, and subsequently the implementation of containment interventions. Delays in the provision of official guidance on camel surveillance and laboratory testing were even seen to decrease interest from national animal health authorities and field workers to implement a large-scale outbreak operation similar to the one executed by the public health sector [48]. It is likely that the response time would have been different if MERS-CoV also had caused significant disease in the animals.

The identification of outbreak milestones also allows for the comparison of timeliness across epidemics. When comparing the MERS (2012), severe acute respiratory syndrome (SARS) (2002), and COVID-19 (2019) outbreaks, which were all epidemics caused by an emerging coronavirus, some differences are notable. To allow this comparison, a comprehensive report from the National Academy of Sciences was used to identify milestones and their execution date for the SARS epidemic [3], and the official WHO timeline was used to identify milestones for COVID-19 [49]. The timeliness of reaching some milestones clearly improved over time. The milestones concerning laboratory confirmation, developing a case definition, sharing the first viral genomic sequence data (microbiological investigation) and developing the first diagnostic assay were all reached much faster during each subsequent epidemic. These improvements are mostly attributed to technological and infrastructural developments, improved response capacity and public health systems, and accumulation of knowledge and experiences acquired from past epidemics [11,13,50]. For the SARS epidemic, for instance, it took about 5 months from the detection of patient zero until the causative agent was identified and its sequence data were shared [3,4]. For MERS, this timeline reduced to 1 month [30]. In sharp contrast, for COVID-19, 2 weeks after the identification of the first cases, the causative agent had been isolated, genetically sequenced and a diagnostic test developed [4,49]. This historical review thereby adds a quantitative benchmark to the ‘as soon as possible’ that is dictated by current convention.

For pathogen detection, and the following related milestones such as notification and reporting, and publication of guidelines in case finding and management, the perception of timeliness is very much dependent on disease specific characteristics such as level of transmissibility, routes of transmission and disease severity [2,9-11]. For instance, understanding MERS-CoV transmission took longer than for the other epidemics, most probably due to its limited human-to-human transmission and the important role of camels, which can be more difficult to detect than when there are high rates of human-to-human transmission as in the case of SARS and COVID-19. Timeliness for early detection and reporting is often defined based on indicators such as identification before detection in secondary cases, before stable human-to-human transmission and before transmission extends from a local/national level to a regional/global level [7]. For notification and reporting, timeliness is perceived as a signal as soon as there is information about a suspected, probable or confirmed case, with the IHR defining a time limit for notification of 24 h after event detection [8,24].

The publication of the first evidence of a potential intermediary animal host for MERS-CoV also took significantly longer than was the case for SARS (6 months from the outbreak detection), even though the importance of zoonotic transmission was much higher [3]. For COVID-19, 3 months after its detection, pangolins were identified as a potential intermediary host with relevance for zoonotic transmission, and later other animal populations tested positive for severe acute respiratory syndrome coronavirus 2 (SARS-CoV-2), such as minks, dogs, domestic cats, lions and tigers [49]. Over 1 year into the COVID-19 pandemic, there is still no strong evidence indicating an intermediary host of importance for sustained zoonotic transmission, although the hypothesis that the virus emerged in a wet market in Wuhan, China through zoonotic transmission is widely supported [51]. In fact, when considering investigations on potential environmental and animals hosts of EZD, most of the knowledge building process is incremental, supported by previous studies done in similar viruses. For instance, the investigations that started on bats and civets for SARS were revisited when MERS appeared, and rapidly undertaken for COVID-19 [51]. However, for all these EZD, the identification of intermediary hosts was among the slowest milestones executed, and until now there is no clear understanding of the exact routes of zoonotic transmissions.

Finally, while the SARS pandemic was contained in less than 8 months from its onset, MERS continues to persist at the time of publication (8 years from its onset) without an approved treatment or vaccine. Aside from specific diagnostic tests, the response to the MERS epidemic has been dependent on generic countermeasures, limited to the repurposing of compounds already licensed or in development for other diseases, especially those applied for SARS. Vaccine candidates targeting MERS-CoV have failed to progress further than early stages of research and development (R&D) because of lack of widespread interest from funders and industrial support [21]. Although the aim for MERS has not yet been achieved, through the partnerships within the WHO’s R&D Blueprint strategy and the Coalition for Epidemic Preparedness Innovations (CEPI), the vaccine development for the current COVID-19 pandemic was executed before 1 year from the epidemic onset – which is the fastest timeline ever achieved [50,52]. Importantly, this also highlights that outbreak R&D preparedness not only supports immediate response measures but also long-term capacity, as lessons learned, and antiviral and vaccine candidates developed against MERS and SARS are now being assessed for repurposing towards COVID-19 [5].

This MERS historical review has some limitations. The review was based primarily on information published on the websites from the Tripartite Partnership for One Health organisations, which are official and reliable sources of information. Nevertheless, at times, available archives did not date back to the beginning of the epidemic. The snowball sampling method was applied to decrease this limitation by identifying complementary data sources. Inherent limitations of public data sources are also relevant, as outbreak information can be shared through private channels or executed earlier and reported only in a later date. In addition, reporting biases could be linked to the inclusion of sources restricted to English language and the international scope of publication. Another critical point regarding the analysis in this paper is that it is focused on assessing timeliness for the responses at the global coordination (supranational) level. National responses, although highly influenced by the supranational guidance and support, had their own specific timelines that are not included in this review. Finally, MERS is a zoonosis that is not associated with significant disease in animals, which directly influenced the types of milestones executed in the animal health domain and their timeliness. Future studies, focused on zoonoses with direct animal health relevance, could help to better identify and investigates milestones in this domain. Despite these limitations, this is the first study to evaluate timeliness in the execution of critical response steps (as outbreak milestones) at the international level for an EZD throughout all epidemic phases and considering the response at the animal–human interface.

## Conclusions

Defining outbreak milestones is important to bring a better understanding on their execution process, timeliness, as well as opportunities and challenges. This historical review identified clear improvements over time in timeliness for the execution of certain milestones including laboratory confirmation and diagnostics development, when comparing the three coronavirus epidemics, i.e. MERS (2012), SARS (2002) and COVID-19 (2019). However, it also identified critical delays in milestone execution for MERS and indicated steps for which no clear improvement was noted when comparing with SARS and COVID-19, such as the identification of intermediary hosts with zoonotic relevance. Future studies should focus on understanding why these delays occurred, and as inputs in the development of meaningful solutions. Further assessments of timeliness can also focus on national response efforts and comparing the different outbreaks including the current COVID-19 pandemic. It is of foremost importance that these delays are widely assessed and tackled in order to more efficiently respond to EZD threats. To achieve this, more complete and precise reporting of outbreak milestones and their execution date is needed. In addition, addressing challenges in the sharing of information, data and resources is crucial.

## Supplementary Data

1290000 Supplement
