# Supplementary material for: A framework for measuring timeliness in the outbreak response path: lessons learned from the Middle East respiratory syndrome (MERS) epidemic, September 2012 to January 2019
Source: Euro Surveill. 2022 Dec 1;27(48):2101064. doi: 10.2807/1560-7917.ES.2022.27.48.2101064 (PMC9716647; doi:10.2807/1560-7917.ES.2022.27.48.2101064)
Supplement: Supplement [file 21-01064_RIBEIRO_SUPPLEMENT.pdf]

S1. Description of the MERS analytical timeline for the phases: alert, I and II epidemic, transition, and enzootic

This supplementary material is hosted by Eurosurveillance as supporting information alongside the article [A framework for measuring timeliness in the outbreak response path: lessons learned from the Middle East respiratory syndrome (MERS) epidemic, September 2012 to January 2019], on behalf of the authors, who remain responsible for the accuracy and appropriateness of the content. The same standards for ethics, copyright, attributions and permissions as for the article apply. Supplements are not edited by Eurosurveillance and the journal is not responsible for the maintenance of any links or email addresses provided therein.

The references (between brackets) in the text of the Supplementary Material S1 are described in the Supplementary Table S1.1. Descriptive list of the literature reviewed for the construction of the analytical timeline divided in types of literature.

Alert phase: MERS-CoV detection and primary response (Supplementary Figure S1.1)

Initial detection of MERS-CoV emergence occurred through the health care system, when patient zero (P0) was admitted to a private hospital in Saudi Arabia (Milestone 1) [28]. Factors that played a role in the delays for MERS-CoV detection were firstly the process of excluding known causative agents, and the time for ordering and receiving the diagnostic kits needed for this process [29]. In order to confirm his diagnosis, the local virologist shipped a (blood) sample from P0 abroad, to a laboratory in the Netherlands. Three months after the hospitalization of P0, the diagnosis of a novel betacoronavirus species was confirmed (Milestone 2) [28,29]. The local virologist submitted the first report on the discovery of this new pathogen MERS-CoV to ProMED mail a few days after receiving the confirmation (Milestone 3) [29]. Two days after this alert, the United Kingdom public health agency, notified the World Health Organization (WHO) of a new human case from a patient travelling from Qatar. WHO alerted all its Member States about the emergence of the new coronavirus, and activated the notification and reporting system under the International Health Regulations (IHR) [30]. Three days later, WHO issued an interim case definition to standardize and help countries with epidemic investigations and development of surveillance activities (Milestone 4) [30].

Initial response measures implemented to support containment efforts were the development and publication of the first diagnostic method for MERS-CoV (Milestone 5) that was enabled by the sharing of the first genetic sequence data (GSD) from P0 [31]. This diagnostic method was made publicly available, and - under a laboratory network coordinated by WHO – support was provided for its production and distribution to affected countries to enable case finding [30,32]. Two months later, WHO provided the first surveillance recommendations for MERS-CoV human infections, by advising countries to use their existing surveillance systems to identify and test all patients with unexplained pneumonias (Milestone 6) [30,33]. Four months after that, WHO published the first guidance on the clinical management of human cases (Milestone 8) [34]. Efforts on the international investigation of cases started with WHO missions to affected countries aiming to support their response strategies and gather data for a better understanding of the epidemic, including transmission routes [30]. The first suspicion of potential human-to-human transmission of MERS-CoV was voiced in October 2012, with the retrospective analysis of samples from a hospital outbreak in Jordan that happened in April 2012, showing viral match with the MERS-CoV [32]. From investigations of a MERS cluster in London, a new case was found without travel history to the Middle East, providing evidence of human-to-human transmission (Milestone 7). Based on the accumulated evidence from the Arabian Peninsula and Europe, showing rare community and household transmissions [3], there was strong indication that human-to-human transmission existed but was limited [30,35].

At this phase, investigations on the molecular characterization of the MERS-CoV started across the globe, enabled by the publication of the first viral GSD (from P0) (Milestone 9). This was closely followed by the publication of the first epidemiological and clinical investigations of P0 (Milestone 10) [28]. The investigations of potential animal and/or environmental sources of MERS-CoV were initiated in the same period when samples from bats in the Bisha area of Saudi Arabia were collected and tested in the United States, showing a viral match with MERS-CoV (Milestone 9) [32,36]. In addition, the potential involvement of camels as an intermediary source of MERS-CoV infection in humans was considered early in the epidemic (September 2012), due to epidemiological investigations indicating close contact of human cases with camels and sheep (Milestone 11) [32]. Finally, in

January 2013, WHO organized the first international meeting on MERS, to perform a risk assessment involving affected countries and international experts, as well as to develop a gap analysis and identify next steps in the global response (Milestone 12) [32].

I Epidemic phase: detection in camels and implementation of containment, control and mitigation strategies (Supplementary Figure S1.2)

In August 2013, scientists first reported findings of a serological studies showing that camels had antibodies against MERS-CoV (Milestone 1) [37-43], closely followed by the laboratory confirmation detecting the virus in camels linked to confirmed human cases (Milestone 2) [44-49]. Both findings (antibodies and the virus) were reported to the international community, through WHO, before the scientific findings were published in peer-reviewed journals, which decreased the timeline for informing response measures [30,74]. A belated serological survey of camels from Saudi Arabia revealed that MERS-CoV has been circulating in these animals and countrywide since at least 1992 [41], with camels being asymptomatic or exhibiting only mild symptoms [73].

With the performance of the first serological and epidemiological studies in animals (Milestone 10), the validation of a serologic assay for specific use in camels was also achieved (Milestone 5) [37]. Following these scientific outcomes, on November 2013, WHO for the first time advised the uptake of hygiene measures to prevent zoonotic infections focused on people at high risk of severe disease and in close contact with camels (Milestone 13) [30]. Four months later, the WHO published a statement on its website recognizing camels as a source of human infections (Milestone 7) [50]. The first global advise on infection prevention and control (IPC) measures targeted at nosocomial transmissions was published on May 2013 (Milestone 13) [51]. The delay in developing the guidance on IPC measures was mostly related to the dependence on the understanding of how transmission takes place. This followed the evidence that MERS could be acquired in health care facilities, as it was the first-time health care workers were diagnosed with MERS-CoV after exposure to patients [30].

Following the recognition of zoonotic transmission, it was organized the first international meeting engaging the Tripartite partnership for One Health (OH) engaging the following organizations: WHO, FAO (Food and Agriculture Organization) and WOAH (World Organization for Animal Health, previously named and identified in the Supplementary Figures S1-5 and Supplementary Tables S1-3 as OIE). It took place in December 2013, in Egypt, representing the development of the first joined risk assessment at the animal human interface (Milestone 12). In addition, it also represented the first global effort to coordinate multinational studies (case-control and sero-epidemiological) for human and animal infections to address critical knowledge gaps surrounding the transmission route of MERS-CoV, and help in determining risk factors that present opportunities for transmission (Milestone 14) [52]. In a next meeting, five months later in Oman, global advice for animal surveillance and management of MERS-CoV positive camels was given in the Muscat Declaration (Milestone 6) [53].

II Epidemic phase: containment, control and mitigation also at the animal-human interface (Supplementary Figure S1.3)

With the confirmation of camels as a source of MERS-CoV zoonotic transmission, on July 2014 the WOAH recognized MERS as an emerging disease in camels with zoonotic potential, and requested countries to notify and report positive camels (Milestone 3) [54]. This notification occurred after deliberations on whether MERS fulfilled the criteria of a notifiable disease; since MERS-CoV does not cause significant disease in animals it cannot be included in the OIE (WOAH) List of Diseases, while at the same time laboratory and epidemiological evidence supported the inference of zoonotic transmission [22].

The WOAH expert group also made recommendations for developing a case definition for camel infections, considered as PCR positive animals (Milestone 4). To support case finding and management, experts noted that validation of the available (PCR) diagnostics for animal surveillance according to the WOAH standards was still needed [54]. Further guidance for the implementation of camel surveillance and management of positive cases was provided in April 2015, in a OH international meeting organized in Qatar [55].

In line with IPC measures, WHO developed a guidance to avoid MERS-CoV transmission from animals to humans with the support from WOAH [54]; and one year after that, stated on its MERS website that stopping zoonotic transmission of the virus was the only way to mitigate nosocomial outbreaks [56]. Meanwhile, the publication of many scientific studies regarding epidemiological, virologic, clinical and environmental

investigations, enhanced knowledge regarding human as well as animal MERS-CoV infections. One meta-study showed however, that the majority of publications on epidemiology of MERS outbreaks (from 2013 to 2015 in Al-Hasa, Jeddah and Riyadh) were performed after the outbreaks had ended [16]. Throughout the epidemic, delays in official notification and reporting for human cases were noticed, when countries struggled to timely notify all new cases and to report detailed epidemiological and clinical information on confirmed and suspected cases, which were essential for updating risk assessment, case definition, guidance for treatment, and case management [30,68,69]. One study mentioned specifically that detailed epidemiological data on non-human exposures, such as direct or indirect animal exposure, was limited for a long period of time to only a few case-control studies [63]. Finally, in October 2015, globally coordinated efforts to perform multinational animal studies were initiated through funding from the United States Agency for International Development (USAID) [57].

Transition phase: the push for globally coordinated investigation, research and development (Supplementary Figure S1.4)

At this phase, most milestones identified for the MERS epidemic were executed, although even until today, no vaccines and therapeutics have been available to counteract the epidemic. After the activation of the notification and reporting of animal cases, the WOAH started publishing country reports; and on May 2017, they published an official case definition for reporting confirmed and suspected camel infections [58], which supported countries to implement and execute animal surveillance and investigations. Among other factors, the late guidance for identification and collection of data on camel infections hindered the control of zoonotic spillovers and further nosocomial outbreaks. The transition phase was also marked by additional efforts to coordinate research and development (R&D) strategies at the international level to move the development of targeted medical countermeasures against MERS-CoV forward. In December 2015, MERS was considered a priority disease under the WHO's Blueprint strategy [59], and five months later, the first roadmap for MERS R&D was published [24]. One year later, the first Target Product Profile for MERS-CoV vaccines was published by WHO followed by more support and funding from the Coalition for Epidemic Preparedness and Innovation (CEPI), to push forward vaccine development [60]. With this push for R&D, many animal studies and clinical trials were performed to test therapeutics and vaccines, besides other epidemiological and clinical evaluation on human and animals.

Enzootic phase: continued zoonotic transmission and emergence of cases (Supplementary Figure S1.5)

Containment efforts are described to support continuous case finding and management though surveillance, cross sectorial investigations and sharing of information. In addition, the implementation of IPC measures, especially targeted at mass gathering events in the Arabian Peninsula region, were noted. Finally, progress in R&D efforts positively marked this phase [61]. Studies published at this phase helped in the understanding and management of nosocomial outbreaks [62], the geographic scope of MERS-CoV circulation in camels, and provided an overview of the amount of zoonotic transmission taking place [63].

Although a decrease in the number and scale of MERS outbreaks is featured in this phase, it is also marked by the constant emergence of community cases due to zoonotic transmission. One study showed that contact with dromedaries likely played an important role in the continued introduction of MERS-CoV into the human population by revealing that among all cases reported to the WHO between January 2015 and mid-April 2018, which were primary cases, over half of them had a zoonotic link [63]. Additionally, persistent knowledge gaps are still present regarding the extent of MERS-CoV circulation and the mechanisms of transmission within dromedary camel herds. A complete understanding of factors related to zoonotic transmission and differences in circulating strains is also lacking. These gaps, added to the lack of medical countermeasures, made it difficult to implement efficient response measures to stop zoonotic transmission and therefore avoid new human infections [61].

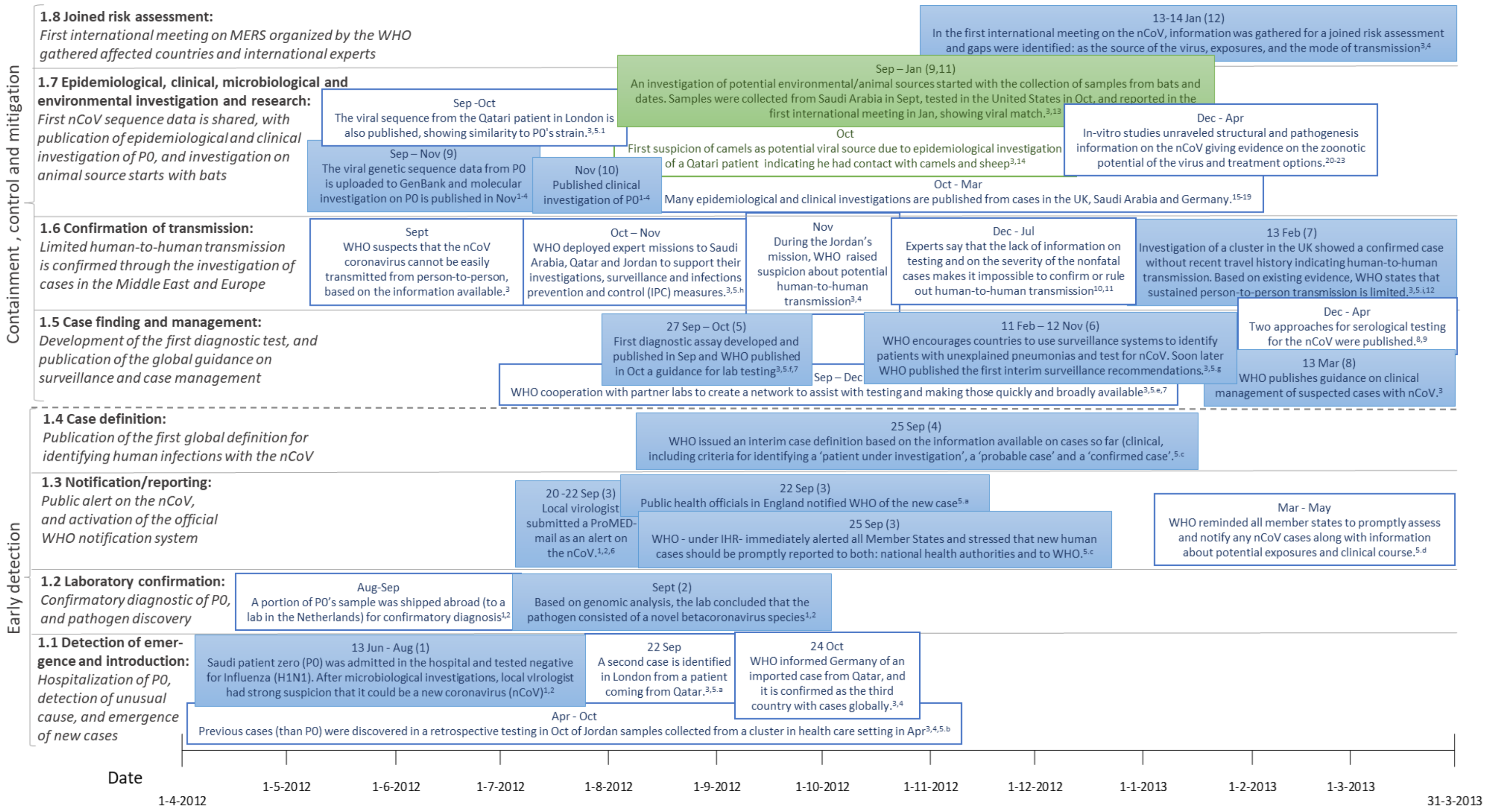

Figure S1.1. Alert phase: MERS-CoV detection and primary response, April 2012 - March 2013 (n=12 Milestones). It is marked by the MERS-CoV detection and implementation of early containment measures.

The timeline is depicted in the bottom, with response events chronologically positioned and clustered according to the type of response intervention they relate to. Events related to the response at the public health domain (focused exclusively on human health) are depicted within blue boxes, while events related to the response at the animal-human interface (also involving the animal health domain) are depicted in green boxes. Full (colored) boxes represent events that mark the execution of outbreak milestones numbered according to Fig. 2 in the main manuscript. The references used in the timeline, as well as reported meetings organized and guidelines published can be found in Supplementary Tables S1.1-3.

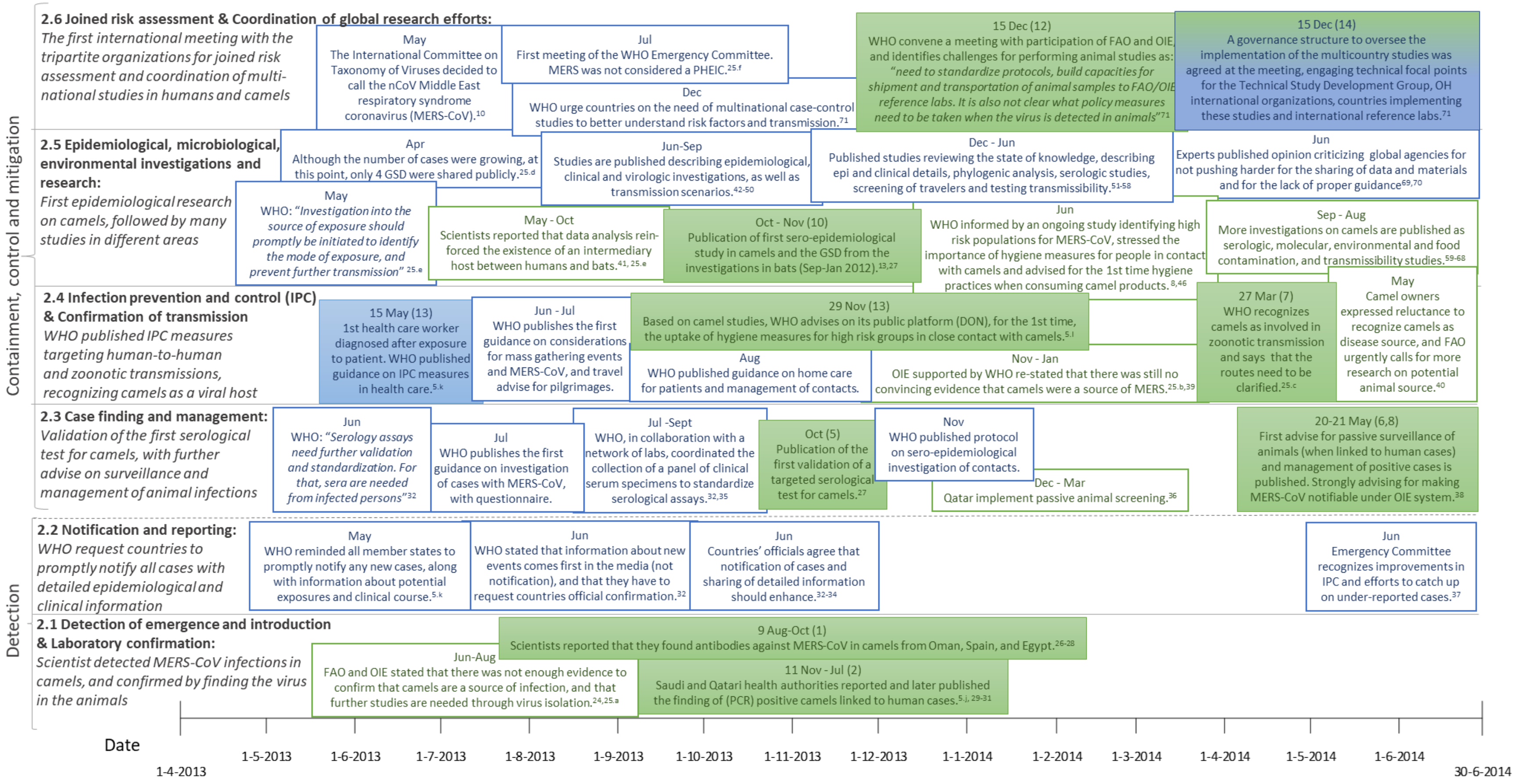

Figure S1.2. I Epidemic phase: detection in camels and implementation of containment, control and mitigation strategies, April 2013 - June 2014 (n=10 Milestones). It is marked by increasing nosocomial outbreaks and the discovery of dromedary camels as an intermediary source of infection.

The timeline is depicted in the bottom, with response events chronologically positioned and clustered according to the type of response intervention they relate to. Events related to the response at the public health domain (focused exclusively on human health) are depicted within blue boxes, while events related to the response at the animal-human interface (also involving the animal health domain) are depicted in green boxes. Full (colored) boxes represent events that mark the execution of outbreak milestones numbered according to Fig. 2 in the main manuscript. The references used in the timeline, as well as reported meetings organized and guidelines published can be found in Supplementary Tables S1.1-3.

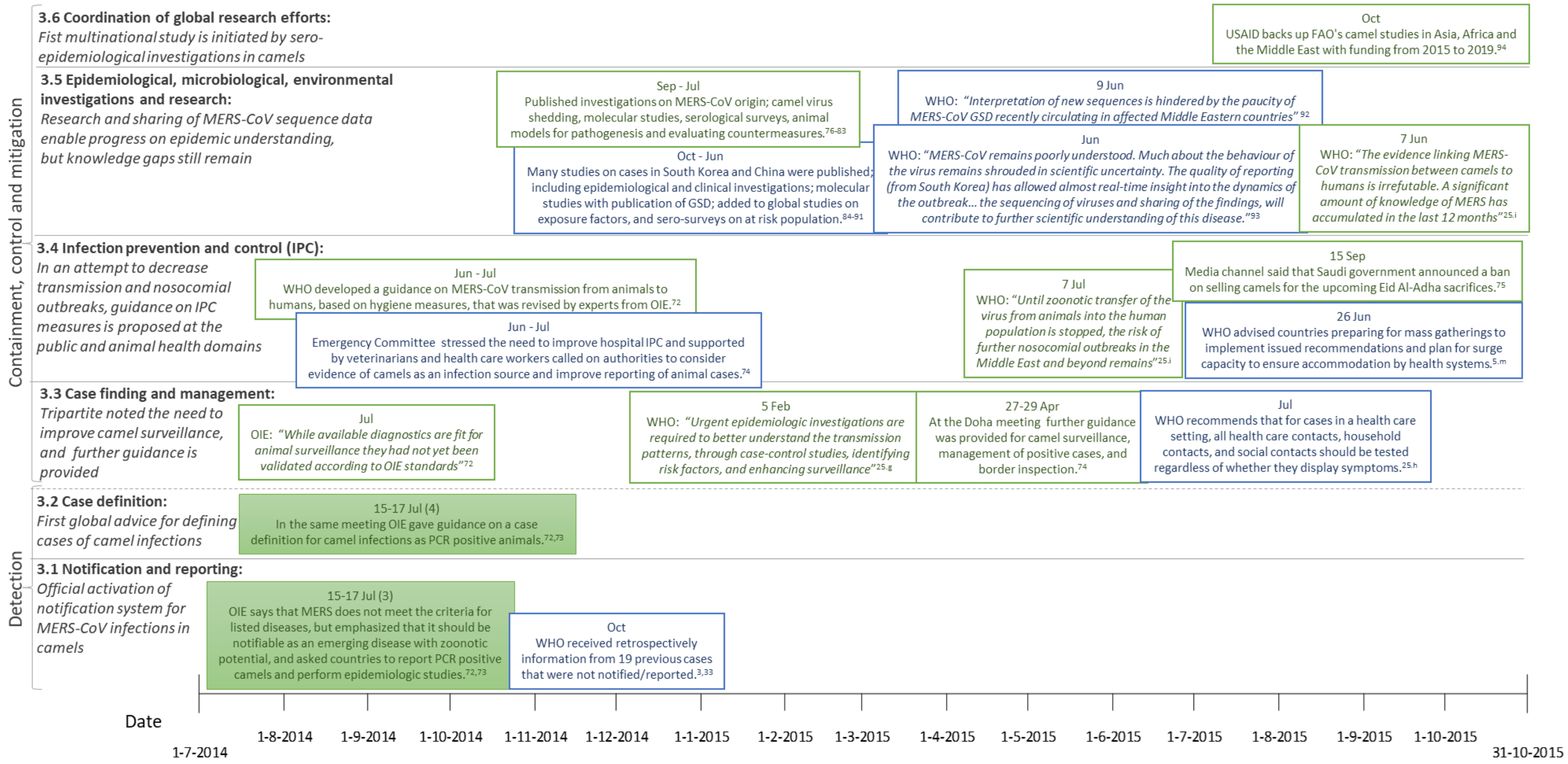

Figure S1.3. II Epidemic phase: containment, control and mitigation also at the animal-human interface, July 2014 - October 2015 (n=2 Milestones). It is marked by the generalized recognition of camels as a MERS-CoV source of infection, and the implementation of more robust response measures at the animal-human interface.

The timeline is depicted in the bottom, with response events chronologically positioned and clustered according to the type of response intervention they relate to. Events related to the response at the public health domain (focused exclusively on human health) are depicted within blue boxes, while events related to the response at the animal-human interface (also involving the animal health domain) are depicted in green boxes. Full (colored) boxes represent events that mark the execution of outbreak milestones numbered according to Fig. 2 in the main manuscript. The references used in the timeline, as well as reported meetings organized and guidelines published can be found in Supplementary Tables S1.1-3.

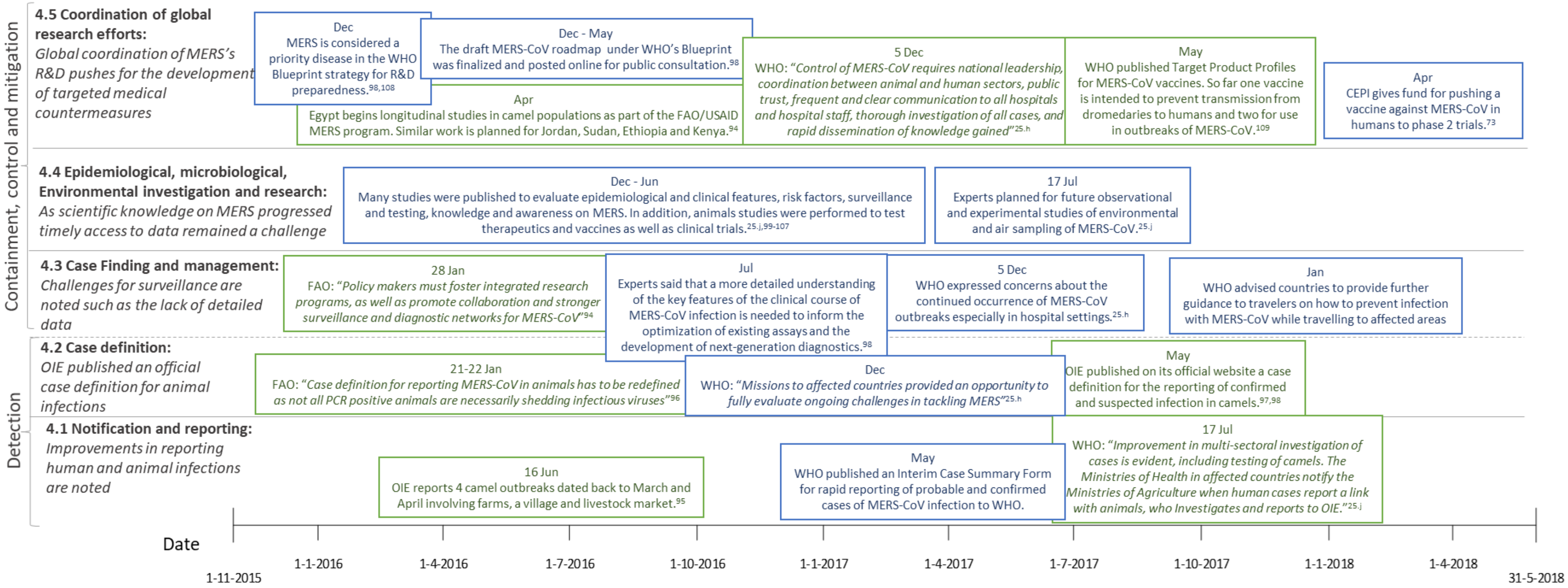

Figure S1.4. Transition phase: the push for globally coordinated investigation, research and development, November 2015 - May 2018. It is marked by the initiation efforts to move forward the development of targeted medical countermeasures as therapeutics and vaccines.

The timeline is depicted in the bottom, with response events chronologically positioned and clustered according to the type of response intervention they relate to. Events related to the response at the public health domain (focused exclusively on human health) are depicted within blue boxes, while events related to the response at the animal-human interface (also involving the animal health domain) are depicted in green boxes. Full (colored) boxes represent events that mark the execution of outbreak milestones numbered according to Fig. 2 in the main manuscript. The references used in the timeline, as well as reported meetings organized and guidelines published can be found in Supplementary Tables S1.1-3.

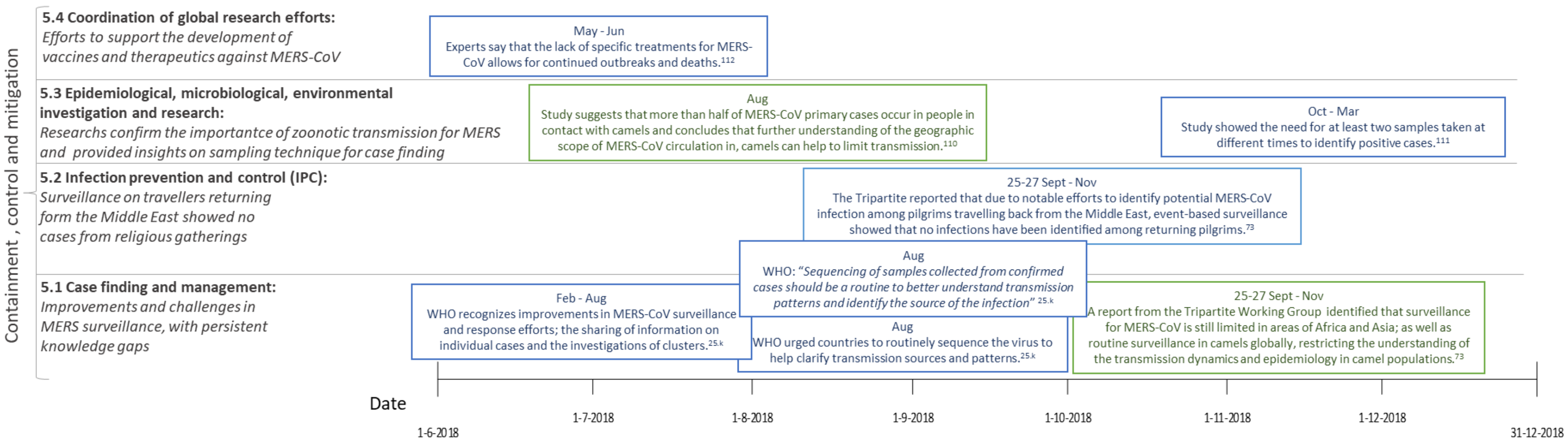

Figure S1.5. Enzootic phase: continued zoonotic transmission and emergence of cases, June - December 2018. It is marked by the constant emergence of human cases and persistent knowledge gaps.

The timeline is depicted in the bottom, with response events chronologically positioned and clustered according to the type of response intervention they relate to. Events related to the response at the public health domain (focused exclusively on human health) are depicted within blue boxes, while events related to the response at the animal-human interface (also involving the animal health domain) are depicted in green boxes. Full (colored) boxes represent events that mark the execution of outbreak milestones numbered according to Fig. 2 in the main manuscript. The references used in the timeline, as well as reported meetings organized and guidelines published can be found in Supplementary Tables S1.1-3.

Detailed description of the literature and data sources

Table S1.1. Descriptive list of the literature reviewed for the construction of the analytical timeline divided in types of literature: peer-reviewed and non-peer-reviewed (grey) literature sources, April 2012 – December 2018 (n=112 sources). The reference number correspond to the ones used in Supplementary Material S1 (between brackets) and Supplementary Figures S1.1-5 (superscript).

Reference Number & Authors	Type of Literature & Source	Publication Date	Title
1. Zaki AM, van Boheemen S, Bestebroer TM, Osterhaus ADME, Fouchier R a. M.	Peer-reviewed Lit.: The New England Journal of Medicine	published on Oct 2012, updated on Jul 2013	Isolation of a Novel Coronavirus from a Man with Pneumonia in Saudi Arabia
2. Hussein I.	Grey Lit. Newsletter Website: Nature Middle East	Jun 2014	The story of the first MERS patient. Interview
3. World Health Organization (WHO).	Grey Lit. Official Website of Governments and International Organizations: WHO-EM/CSR/059/E	14–15 Jan 2013	Report on the technical consultative meeting on novel coronavirus, Cairo, Egypt
4. Joseph C, Malik M, Mounts A, Mafi A, Briand S, Memish Z.	Peer-reviewed Lit: EMHJ-Eastern Mediterranean Health Journal	2013	Highlights and conclusions from the technical consultative meeting on novel coronavirus infection, Cairo, Egypt, 14-16 January 2013
5. World Health Organization (WHO).	Grey Lit. Official Website of Governments and International Organizations: WHO website	5.a – 23 Sep 2012<br>5.b – 30 Nov 2012<br>5.c – 25 Sep 2012<br>5.d – 6 Mar 2013<br>5.e – 28 Sep 2012<br>5.f – 29 Sep 2012<br>5.g – 11 Feb 2013<br>5.h – 10 Oct 2012<br>5.i – 13 Feb 2013<br>5.j – 29 Nov 2013<br>5.k - 15 May 2013<br>5.l – 29 Nov 2013<br>5.m – 26 Jun 2015	MERS-CoV disease outbreak news
6. Zaki AM.	Grey Lit. Surveillance Website: ProMED-mail	20 Sep 2012	Novel coronavirus - Saudi Arabia: human isolate
7. Corman VM, Eckerle I, Bleicker T, Zaki A, Landt O, Eschbach-Bludau M, et al.	Peer-reviewed Lit.: Eurosurveillance	Sep 2012	Detection of a novel human coronavirus by real-time reverse-transcription polymerase chain reaction
8. Corman VM, Müller MA, Costabel U, Timm J, Binger T, Meyer B, et al.	Peer-reviewed: Eurosurveillance	6 Dec 2012	Assays for laboratory confirmation of novel human coronavirus (hCoV-EMC) infections
9. Reusken C, Mou H, Godeke GJ, van der Hoek L, Meyer B, Müller MA, et al.	Peer-reviewed: Eurosurveillance	4 Apr 2013	Specific serology for emerging human coronaviruses by protein microarray
10. de Groot RJ, Baker SC, Baric RS, Brown CS, Drosten C, Enjuanes L., et al.	Peer-reviewed Lit.: Journal of Virology	Jul 2013	Commentary: Middle East Respiratory Syndrome Coronavirus (MERS-CoV): Announcement of the Coronavirus Study Group
11. Center for Infectious Disease Research and Policy (CIDRAP).	Grey Lit. Newsletter Website: CIDRAP	Dec 2012	News and perspective_MERS-CoV

12. Health Protection Agency (HPA) UK Novel Coronavirus Investigation team.	Peer-reviewed Lit.: Eurosurveillance	Feb 2013	Evidence of person-to-person transmission within a family cluster of novel coronavirus infections, United Kingdom, February 2013
13. Memish ZA, Mishra N, Olival KJ, Fagbo SF, Kapoor V, Epstein JH, et al.	Peer-reviewed Lit.: Emerging infectious diseases	Nov 2013	Middle East respiratory syndrome coronavirus in bats, Saudi Arabia
14. Reusken CB, Haagmans BL, Müller MA, Gutierrez C, Godeke GJ, Meyer B, et al.	Peer-reviewed Lit.: The Lancet infectious diseases	Oct 2013	Middle East respiratory syndrome coronavirus neutralising serum antibodies in dromedary camels: a comparative serological study
15. Pebody RG, Chand MA, Thomas HL, Green HK, Boddington NL, Carvalho C, et al.	Peer-reviewed Lit.: Eurosurveillance	4 Oct 2012	The United Kingdom public health response to an imported laboratory confirmed case of a novel coronavirus in September 2012
16. Bermingham A, Chand MA, Brown CS, Aarons E, Tong C, Langrish C, et al.	Peer-reviewed Lit.: Eurosurveillance	4 Oct 2012	Severe respiratory illness caused by a novel coronavirus, in a patient transferred to the United Kingdom from the Middle East, September 2012
17. AlBarrak AM, Stephens GM, Hewson R, Memish ZA.	Peer-reviewed: Saudi Med J	2012	Recovery from severe novel coronavirus infection.
18. Buchholz U, Müller MA, Nitsche A, Sanewski A, Wevering N, Bauer-Balci T, et al.	Peer-reviewed Lit.: Eurosurveillance	21 Feb 2013	Contact investigation of a case of human novel coronavirus infection treated in a German hospital, October-November 2012
19. Health Protection Agency (HPA) UK Novel Coronavirus Investigation team.	Peer-reviewed Lit.: Eurosurveillance	14 Mar 2013	Evidence of person-to-person transmission within a family cluster of novel coronavirus infections, United Kingdom, February 2013
20. Munster VJ, De Wit E, Feldmann H.	Peer-reviewed: The New England journal of medicine	3 Apr 2013	Pneumonia from human coronavirus in a macaque model
21. Müller MA, Raj VS, Muth D, Meyer B, Kallies S, Smits SL, et al.	Peer-reviewed: MBio	11 Dec 2012	Human coronavirus EMC does not require the SARS-coronavirus receptor and maintains broad replicative capability in mammalian cell lines
22. Kindler E, Jónsdóttir HR, Muth D, Hamming OJ, Hartmann R, Rodriguez R., et al.	Peer-reviewed: MBio	19 Feb 2013	Efficient replication of the novel human betacoronavirus EMC on primary human epithelium highlights its zoonotic potential
23. Falzarano D, De Wit E, Martellaro C, Callison J, Munster VJ, et al.	Peer-reviewed: Scientific reports	18 Apr 2013	Inhibition of novel β coronavirus replication by a combination of interferon-α2b and ribavirin
24. World Organisation for Animal Health (OIE).	Grey Lit. Official Website of Governments and International Organizations: OIE website	22 Jul 2013	Questions & Answers on Middle East Respiratory Syndrome Coronavirus (MERS-CoV)
25. Food and Agriculture Organisation of the United Nations (FAO).	Grey Lit. Official Website of Governments and International Organizations: FAO website	9 Aug 2013	Media News
26. World Health Organization (WHO).	Grey Lit. Official Website of Governments and International Organizations: WHO website	25.a - 13 Aug 2013<br>25.b – 20 Jan 2014<br>25.c – 27 Mar 2014<br>25.d – 25 Apr 2013<br>25.e - 8 May 2013<br>25.f – 18 Jul 2013<br>25.g – 5 Feb 2015<br>25.h - 5 Dec 2016<br>25.i – 7 Jul 2015<br>25.j – 17 Jul 2017<br>25.k – Aug 2018	Middle East respiratory syndrome coronavirus (MERS-CoV) summary and literature update
27. Reusken CB, Haagmans BL, Müller MA, Gutierrez C, Godeke GJ, Meyer B, et al.	Peer-reviewed: The Lancet infectious diseases	Oct 2013	Middle East respiratory syndrome coronavirus neutralising serum antibodies in dromedary camels: a comparative serological study

28. Perera RA, Wang P, Gomaa MR, El-Shesheny R, Kandeil A, Bagato O, et al.	Peer-reviewed Lit.: Eurosurveillance	5 Sept 2013	Seroepidemiology for MERS coronavirus using microneutralisation and pseudoparticle virus neutralisation assays reveal a high prevalence of antibody in dromedary camels in Egypt, June 2013
29. Ministry of Health from the Kingdom of Saudi Arabia	Grey Lit. Official Website of Governments and International Organizations: Press release	11 Nov 2013	A New Way Discovered to Recognize the Source of Novel Coronavirus
30. Azhar EI, El-Kafrawy SA, Farraj SA, Hassan AM, Al-Saeed MS, Hashem AM, & Madani, et al.	Peer-reviewed: New England Journal of Medicine	26 Jun 2014	Evidence for camel-to-human transmission of MERS coronavirus
31. Haagmans BL, Al Dhahiry SH, Reusken CB, Raj VS, Galiano M, Myers R, et al.	Peer-reviewed: The Lancet infectious diseases	Feb 2014	Middle East respiratory syndrome coronavirus in dromedary camels: an outbreak investigation
32. World Health Organization (WHO).	Grey Lit. Official Website of Governments and International Organizations: WHO-EM/CSR/063/E	20–22 Jun 2013	Report on the intercountry meeting on the Middle East Respiratory Syndrome Coronavirus [MERS-CoV] outbreak in the Eastern Mediterranean Region, Cairo, Egypt
33. Perl TM, & Price CS.	Peer-reviewed Lit.: Annals of internal medicine	Aug 2015	Orchestrated scientific collaboration: critical to the control of MERS-CoV
34. Saeed AAB, Abedi GR, Alzahrani AG, Salameh I, Abdirizak F, Alhakeem R, et al.	Peer-reviewed Lit.: Emerging Infectious Diseases	Apr 2017	Surveillance and testing for middle east respiratory syndrome coronavirus, Saudi Arabia, april 2015–february 2016
35. Kelly-Cirino C, Mazzola LT, Chua A, Oxenford CJ, & Van Kerkhove MD.	Peer-reviewed Lit.: BMJ global health	Feb 2019	An updated roadmap for MERS-CoV research and product development: focus on diagnostics
36. Nour M, Alhajri M, Farag E, Al-Romaihi H, Al-Thani M, Al-Marri S, & Savoia E.	Peer-reviewed Lit.: International journal of environmental research and public health	Dec 2017	How Do the First Days Count? A Case Study of Qatar Experience in Emergency Risk Communication during the MERS-CoV Outbreak
37. Dr Keiji Fukuda, WHO’s previous assistant Director-General for Health Security and the Environment.	Grey Lit. Official Website of Governments and International Organizations: WHO website	17 Jun 2014	Transcript of the press briefing
38. FAO and Sultanate of Oman.	Grey Lit. Official Website of Governments and International Organizations: FAO website	20-21 May 2014	Muscat Declaration. Middle East Respiratory Syndrome Coronavirus (MERS-CoV) in Animal Species Regional Technical Consultation Meeting. Muscat, Oman
39. World Organisation for Animal Health (OIE).	Grey Lit. Official Website of Governments and International Organizations: OIE website	15 Nov 2013	Questions & Answers on Middle East Respiratory Syndrome Coronavirus (MERS-CoV)
40. Food and Agriculture Organisation of the United Nations (FAO).	Grey Lit. Official Website of Governments and International Organizations: FAO website	23 May 2014	News article: MERS Coronavirus: Stepped up research into role of camels and other animals urged
41. Ithete NL, Stoffberg S, Corman VM, Cottontail VM, Richards LR, Schoeman MC, et al.	Peer-reviewed Lit.: Emerging Infectious Diseases	Oct 2013	Close Relative of Human Middle East Respiratory Syndrome Coronavirus in Bat, South Africa
42. Guery B, Poissy J, El Mansouf L, Séjourné C, Ettahar N, Lemaire X, et al.	Peer-reviewed: The Lancet	29 Jun 2013	Clinical features and viral diagnosis of two cases of infection with Middle East Respiratory Syndrome coronavirus: a report of nosocomial transmission
43. Memish ZA, Zumla AI, Al-Hakeem RF, Al-Rabeeah AA, Stephens GM.	Peer-reviewed: New England Journal of Medicine	27 Jun 2013	Family cluster of Middle East respiratory syndrome coronavirus infections
44. Assiri A, McGeer A, Perl TM, Price CS, Al Rabeeah AA, Cummings DA, et al.	Peer-reviewed: New England Journal of Medicine	1 Aug 2013	Hospital outbreak of Middle East respiratory syndrome coronavirus
45. Drosten C, Seilmaier M, Corman VM, Hartmann W, Scheible G, Sack S, et al.	Peer-reviewed: The Lancet infectious diseases	Sep 2013	Clinical features and virological analysis of a case of Middle East respiratory syndrome coronavirus infection
46. Cauchemez S, Van Kerkhove MD, Riley S, Donnelly CA, Fraser C, Ferguson NM.	Peer-reviewed: Eurosurveillance	13 Jun 2013	Transmission scenarios for Middle East Respiratory Syndrome Coronavirus (MERS-CoV) and how to tell them apart

47. Breban R, Riou J, Fontanet A.	Peer-reviewed: The Lancet	24 Aug 2013	. Interhuman transmissibility of Middle East respiratory syndrome coronavirus: estimation of pandemic risk
48. Assiri A, Al-Tawfiq JA, Al-Rabeeah AA, Al-Rabiah FA, Al-Hajjar S, Al-Barrak A, et al.	Peer-reviewed: The Lancet infectious diseases	Sept 2013	Epidemiological, demographic, and clinical characteristics of 47 cases of Middle East respiratory syndrome coronavirus disease from Saudi Arabia: a descriptive study
49. Khan K, Sears J, Hu VW, Brownstein JS, Hay S, Kossowsky D, et al.	Peer-reviewed: PLoS currents	17 Jul 2013	Potential for the international spread of Middle East respiratory syndrome in association with mass gatherings in Saudi Arabia
50. Puzelli S, Azzi A, Santini MG, Di Martino A, Facchini M, Castrucci MR, et al.	Peer-reviewed: Eurosurveillance	22 Aug 2013	Investigation of an imported case of Middle East respiratory syndrome coronavirus (MERS-CoV) infection in Florence, Italy, May to June 2013
51. Cotten M, Watson SJ, Kellam P, Al-Rabeeah AA, Makhdoom HQ, Assiri A, et al.	Peer-reviewed: The Lancet	14 Dec 2013	Transmission and evolution of the Middle East respiratory syndrome coronavirus in Saudi Arabia: a descriptive genomic study
52. Cauchemez S, Fraser C, Van Kerkhove MD, Donnelly CA, Riley S, Rambaut A, et al.	Peer-reviewed: The Lancet	Jan 2014	Middle East respiratory syndrome coronavirus: quantification of the extent of the epidemic, surveillance biases, and transmissibility
53. Goh GKM., Dunker AK, Uversky V.	Peer-reviewed: PLoS currents	13 Nov 2013	Prediction of intrinsic disorder in MERS-CoV/HCoV-EMC supports a high oral-fecal transmission
54. Aburizaiza AS, Mattes FM, Azhar EI, Hassan AM, Memish ZA, Muth D, et al.	Peer reviewed: J Infect Dis	15 Jan 2014	Investigation of anti-MERS-coronavirus antibodies in blood donors and abbatoir workers in Jeddah and Makkah, Kingdom of Saudi Arabia, fall 2012
55. Gierer S, Hofmann-Winkler H, Albuali WH, Bertram S, Al-Rubaish AM, Yousef AA, et al.	Peer-reviewed: Emerging infectious diseases	Dec 2013	Lack of MERS coronavirus neutralizing antibodies in humans, eastern province, Saudi Arabia
56. Memish ZA, Cotten M, Meyer B, Watson SJ, Alsahafi AJ, Al Rabeeah AA, et al.	Peer-reviewed: Emerging infectious diseases	Jun 2014	Human infection with MERS coronavirus after exposure to infected camels, Saudi Arabia, 2013
57. Memish ZA, Assiri A, Almasri M, Alhakeem RF, Turkestani A, Al Rabeeah AA, et al.	Peer-reviewed: The Journal of infectious diseases	11 Mar 2014	Prevalence of MERS-CoV nasal carriage and compliance with the Saudi health recommendations among pilgrims attending the 2013 Hajj
58. Kraaij–Dirkzwager M, Timen A, Dirksen K, Gelinck L, Leyten E, Groeneveld P, et al.	Peer-reviewed: Eurosurveillance	29 May 2014	Middle East respiratory syndrome coronavirus (MERS-CoV) infections in two returning travellers in the Netherlands, May 2014
59. Reusken CB, Ababneh M, Raj VS, Meyer B, Eljarah A, Abutarbush S, et al.	Peer-reviewed: Eurosurveillance	12 Dec 2013	Middle East Respiratory Syndrome coronavirus (MERS-CoV) serology in major livestock species in an affected region in Jordan, June to September 2013
60. Meyer B, Müller MA, Corman VM, Reusken CB, Ritz D, Godeke GJ, et al.	Peer-reviewed: Emerging infectious diseases	Apr 2014	Antibodies against MERS coronavirus in dromedary camels, United Arab Emirates, 2003 and 2013
61. Chu DK, Poon LL, Gomaa MM, Shehata MM, Perera RA, Zeid DA, et al.	Peer-reviewed: Emerging infectious diseases	Jun 2014	MERS coronaviruses in dromedary camels, Egypt
62. Alagaili AN, Briese T, Mishra N, Kapoor V, Sameroff SC, de Wit E, et al.	Peer-reviewed: MBio	25 Feb 2014	Middle East respiratory syndrome coronavirus infection in dromedary camels in Saudi Arabia
63. Briese T, Mishra N, Jain K, Zalmout IS, Jabado OJ, Karesh WB, et al.	Peer-reviewed: MBio	29 Apr 2014	Middle East respiratory syndrome coronavirus quasispecies that include homologues of human isolates revealed through whole-genome analysis and virus cultured from dromedary camels in Saudi Arabia
64. van Doremalen N, Bushmaker T, Karesh WB, Munster VJ.	Peer-reviewed: Emerging infectious diseases	Jul 2014	Stability of Middle East respiratory syndrome coronavirus in milk
65. Reusken CB, Messadi L, Feyisa A, Ularamu H, Godeke GJ, Danmarwa A, et al.	Peer-reviewed: Emerging infectious diseases	Aug 2014	Geographic distribution of MERS coronavirus among dromedary camels, Africa

66. Nowotny N, Kolodziejek J.	Peer-reviewed: Eurosurveillance	24 Apr 2014	Middle East respiratory syndrome coronavirus (MERS-CoV) in dromedary camels, Oman, 2013
67. Van Doremalen N, Bushmaker T, Munster VJ.	Peer-reviewed: Eurosurveillance	19 Sep 2013	Stability of Middle East respiratory syndrome coronavirus (MERS-CoV) under different environmental conditions
68. Hemida MG, Chu DK, Poon LL, Perera RA, Alhammadi MA, Ng HY, et al.	Peer-reviewed: Emerging infectious diseases	Jul 2014	MERS coronavirus in dromedary camel herd, Saudi Arabia
69. Nature Editorial.	Peer-reviewed Lit.: Nature	Jun 2014	Present danger_ There is much hype about predicting and preventing future pandemics, but not enough is being done about a threat sitting under our noses
70. McNabb SJ, Shaikh AT, Nuzzo JB, Zumla AI, & Heymann DL.	Peer-reviewed Lit.: The lancet Respiratory medicine	Jun 2014	Triumphs, trials, and tribulations of the global response to MERS coronavirus
71. World Health Organization (WHO).	Grey Lit. Official Website of Governments and International Organizations: WHO-EM/CSR/068/E	15–16 Dec 2013	Summary report on the consultative meeting to determine a public health research agenda on MERS-CoV, Cairo, Egypt
72. World Organisation for Animal Health (OIE).	Grey Lit. Official Website of Governments and International Organizations: OIE website	15-17 Jul 2014	OIE Ad hoc group on MERS-CoV infection in animals. Paris
73. Aguanno R, ElIdrissi A, Elkholy AA, Embarek PB, Gardner E, Grant R, et al.	Peer-reviewed Lit.: Antiviral research	Sep 2018	MERS: Progress on the global response, remaining challenges and the way forward
74. FAO and meeting participants.	Grey Lit. Official Website of Governments and International Organizations: FAO website	27-29 Apr 2015	Doha Declaration. Regional Workshop on MERS-CoV and One Health. Doha, Qatar
74. Dr Keiji Fukuda, WHO's previous assistant Director-General for Health Security and the Environment.	Grey Lit. Official Website of Governments and International Organizations: WHO website	17 Jun 2014	Transcript of the press briefing
75. Undefined	Grey Lit. Newsletter Website: Arab News	15 Sep 2015	MERS fears send camel prices crashing
76. Wang Q, Qi J, Yuan Y, Xuan Y, Han P, Wan Y, et al.	Peer-reviewed: Cell host & microbe	10 Sep 2014	Bat origins of MERS-CoV supported by bat coronavirus HKU4 usage of human receptor CD26
77. Adney DR, van Doremalen N, Brown VR, Bushmaker T, Scott D, de Wit E, et al.	Peer-reviewed: Emerging infectious diseases	Dec 2014	Replication and shedding of MERS-CoV in upper respiratory tract of inoculated dromedary camels
78. Meyer B, García-Bocanegra I, Wernery U, Wernery R, Sieberg A, Müller MA, et al.	Peer-reviewed: Emerging infectious diseases	Jan 2015	Serologic assessment of possibility for MERS-CoV infection in equids
79. Agrawal AS, Garron T, Tao X, Peng BH, Wakamiya M, Chan TS, et al.	Peer-reviewed: Journal of virology	9 Jan 2015	Generation of a transgenic mouse model of Middle East respiratory syndrome coronavirus infection and disease
80. Chan RW, Hemida MG, Kayali G, Chu DK, Poon LL, Alnaeem A, et al.	Peer-reviewed: The Lancet Respiratory Medicine	Oct 2014	Tropism and replication of Middle East respiratory syndrome coronavirus from dromedary camels in the human respiratory tract: an in-vitro and ex-vivo study
81. Müller MA, Corman VM, Jores J, Meyer B, Younan M, Liljander A, et al.	Peer-reviewed: Emerging infectious diseases	Dec 2014	MERS coronavirus neutralizing antibodies in camels, Eastern Africa, 1983–1997
82. Chan SM, Damdinjav B, Perera RA, Chu DK, Khishgee B, Enkhbold B, et al.	Peer-reviewed: Emerging infectious diseases	Jul 2015	Absence of MERS-coronavirus in Bactrian camels, southern Mongolia, November 2014
83. Khalafalla AI, Lu X, Al-Mubarak AI, Dalab AHS, Al-Busadah KA, Erdman DD.	Peer-reviewed: Emerging infectious diseases	Jul 2015	MERS-CoV in upper respiratory tract and lungs of dromedary camels, Saudi Arabia, 2013–2014

84. Fanoy EB, Van Der Sande MA, Kraaij-Dirkzwager M, Dirksen K, Jonges M, Van Der Hoek W, et al.	Peer-reviewed: Emerging themes in epidemiology	17 Oct 2014	Travel-related MERS-CoV cases: an assessment of exposures and risk factors in a group of Dutch travellers returning from the Kingdom of Saudi Arabia, May 2014
85. Memish ZA, Alsahly A, Masri MA, Heil GL, Anderson BD, Peiris M, et al	Peer-reviewed: . Influenza and other respiratory viruses	3 Dec 2014	Sparse evidence of MERS-C o V infection among animal workers living in S outhern S audi A rabia during 2012
86. Park HY, Lee EJ, Ryu YW, Kim Y, Kim H, Lee H, Yi SJ.	Peer-reviewed: Eurosurveillance	25 Jun 2015	Epidemiological investigation of MERS-CoV spread in a single hospital in South Korea, May to June 2015
87. Cowling BJ, Park M, Fang VJ, Wu P, Leung GM, Wu JT.	Peer-reviewed: Eurosurveillance	25 Jun 2015	Preliminary epidemiological assessment of MERS-CoV outbreak in South Korea, May to June 2015
88. Wu J, Yi L, Zou L, Zhong H, Liang L, Song T, et al.	Peer-reviewed: Eurosurveillance	18 Jun 2015	Imported case of MERS-CoV infection identified in China, May 2015: detection and lesson learned
89. Müller MA, Meyer B, Corman VM, Al-Masri M, Turkestani A, Ritz D, et al.	Peer-reviewed: . The Lancet Infectious Diseases	May 2015	Presence of Middle East respiratory syndrome coronavirus antibodies in Saudi Arabia: a nationwide, cross-sectional, serological study
90. Oboho IK, Tomczyk SM, Al-Asmari AM, Banjar AA, Al-Mugti H, Aloraini MS, et al.	Peer-reviewed: New England Journal of Medicine	26 Feb 2015	2014 MERS-CoV outbreak in Jeddah—a link to health care facilities
91. Al-Gethamy M, Corman VM, Hussain R, Al-Tawfiq JA, Drosten C, Memish ZA.	Peer-reviewed: Clinical Infectious Diseases	15 Mar 2015	A case of long-term excretion and subclinical infection with Middle East respiratory syndrome coronavirus in a healthcare worker
92. World Health Organization (WHO).	Grey Lit. Official Website of Governments and International Organizations: WHO website	9 Jun 2015	MER-CoV situation assessment. Preliminary data from sequencing of viruses in the Republic of Korea and the People's Republic of China
93. World Health Organization (WHO).	Grey Lit. Official Website of Governments and International Organizations: WHO website - Media Center	2015	MERS-CoV outbreak largest outside Kingdom of Saudi Arabia
94. Food and Agriculture Organisation of the United Nations (FAO).	Grey Lit. Official Website of Governments and International Organizations: FAO website	28 Jan 2016	News article: FAO pushes for a road map to control and contain zoonotic diseases
95. World Organisation for Animal Health (OIE).	Grey Lit. Official Website of Governments and International Organizations: OIE website	16 Jun 2016	Report MERS-CoV Saudi Arabia
96. Food and Agriculture Organisation of the United Nations (FAO).	Grey Lit. Official Website of Governments and International Organizations: FAO website	21-22 Jan 2016	Report: FAO Technical Meeting on Understanding MERS-CoV at the animal-human interface, Rome, Italy
97. World Organisation for Animal Health (OIE).	Grey Lit. Official Website of Governments and International Organizations: OIE website	May 2017	Middle East Respiratory Syndrome Coronavirus (MERS-CoV) Case Definition for Reporting to OIE
98. Modjarrad K, Moorthy VS, Embarek PB, Van Kerkhove M, Kim J, & Kieny MP.	Peer-reviewed Lit.: Nature medicine	Jul 2016	A roadmap for MERS-CoV research and product development: report from a World Health Organization consultation
99. Mohd HA, Memish ZA, Alfaraj SH, McClish D, Altuwaijri T, Alanazi MS, et al.	Peer-reviewed: Travel medicine and infectious disease	24 Sep 2016	Predictors of MERS-CoV infection: a large case control study of patients presenting with ILI at a MERS-CoV referral hospital in Saudi Arabia
100. Sikkema RS, Farag EA, Himatt S, Ibrahim AK, Al-Romaihi H, Al-Marri SA, et al.	Peer-reviewed: The Journal of infectious diseases	1 Jun 2017	Risk factors for primary Middle East respiratory syndrome coronavirus infection in camel workers in Qatar during 2013–2014: a case-control study
101. Saeed AAB, Abedi GR, Alzahrani AG, Salameh I, Abdirizak F, Alhakeem R, et al.	Peer-reviewed: Emerging infectious diseases	Apr 2017	Surveillance and testing for middle east respiratory syndrome coronavirus, Saudi Arabia, April 2015–February 2016
102. Kang CK, Song KH, Choe PG, Park WB, Bang JH, Kim ES, et al.	Peer-reviewed: Journal of Korean medical science	May 2017	Clinical and epidemiologic characteristics of spreaders of Middle East respiratory syndrome coronavirus during the 2015 outbreak in Korea
103. Moon SY, Son JS.	Peer-reviewed: Clinical Infectious Diseases	15 May 2017	Infectivity of an asymptomatic patient with Middle East respiratory syndrome coronavirus infection

104. Alqahtani AS, Rashid H, Basyouni MH, Alhawassi TM, BinDhim NF.	Peer-reviewed: Journal of Infection and Public Health	18 Nov 2016	Public response to MERS-CoV in the Middle East: iPhone survey in six countries
105. Chan JFW, Yao Y, Yeung ML, Deng W, Bao L, Jia L, et al... & Cai, J. P.	Peer-reviewed: The Journal of infectious diseases	15 Dec 2015	Treatment with lopinavir/ritonavir or interferon-β1b improves outcome of MERS-CoV infection in a nonhuman primate model of common marmoset
106. Agrawal AS, Ying T, Tao X, Garron T, Algaissi A, Wang Y, et al... & Tseng, C. T. K.	Peer-reviewed: Scientific reports	19 Aug 2016	Passive transfer of a germline-like neutralizing human monoclonal antibody protects transgenic mice against lethal Middle East respiratory syndrome coronavirus infection
107. Lan J, Yao Y, Deng Y, Chen H, Lu G, Wang W, et al... & Qin, C.	Peer-reviewed: EBioMedicine	Oct 2015	Recombinant receptor binding domain protein induces partial protective immunity in rhesus macaques against Middle East respiratory syndrome coronavirus challenge
108. World Health Organization (WHO).	Grey Lit. Official Website of Governments and International Organizations: WHO website	2018	WHO R&D Blueprint List of Priority Diseases
109. World Health Organization (WHO).	Grey Lit. Official Website of Governments and International Organizations: WHO website	May 2017	WHO Target Product Profiles for MERS-CoV Vaccines
110. Conzade R, Grant R, Malik MR, Elkholy A, Elhakim M, Samhouri D, et al.	Peer-reviewed Lit.: Viruses	Aug 2018	Reported direct and indirect contact with dromedary camels among laboratory-confirmed MERS-CoV cases
111. Alfaraj SH, Al-Tawfiq JA, & Memish ZA.	Peer-reviewed Lit.: American journal of infection control	Mar 2019	Middle East respiratory syndrome coronavirus intermittent positive cases: implications for infection control
112. National Institutes of Health (NIH).	Grey Lit. Newsletter Website: NIH website – News release	May 2018	Experimental MERS treatments enter clinical trial: NIH-sponsored trial to test two human monoclonal antibodies

Table S1.2. Description of regional and international meetings that took place throughout the epidemic phases (of alert phase; I epidemic phase; II epidemic phase; transition phase; and enzootic phase) addressing the response to MERS-CoV, April 2012 – December 2018 (n=33 meetings).

PHASE	*MEETING*	*PLACE*	*DATE*	*CONTENT*
ALERT PHASE	Technical consultative meeting on novel coronavirus infection	Cairo, Egypt	14-16 Jan 2013	Review and discuss the scientific and public health understanding of the emergence of nCoV to date, identify critical knowledge gaps in understanding the current risk and identify the next steps to improve knowledge and close up the research gap for public health action at the national and international level. In each meeting session, available scientific information and up-to-date evidence on nCoV were presented and discussed by the participants. The sessions included (i) epidemiological information; (ii) virological and animal investigation; (iii) development of tests for nCoV; (iv) experience from SARS; and (v) risk communication and preparedness.
I EPIDEMIC PHASE	WHO Intercountry meeting on the MERS-CoV outbreak in the Eastern Mediterranean Region	Cairo, Egypt	20-22 Jun 2013	Overview and update on the outbreak, country specific updates, technical guidance on surveillance, outbreak and preparedeness for mass gatherings, technical support and interim guidance for cases' management, technical guidnace and support for laboratory diagnostics, guidance on infection prevention and control, reflections on media and risk communication.
	WHO-ISARIC joint MERS-CoV Outbreak Readiness Workshop: Clinical management and potential use of convalescent plasma	Geneva, Switzerland	10-12 Dec 2013	Participants agreed to work towards developing a regional clinical research network and agreed on: 1) conducting a feasibility survey in the countries to identify suitable sites and current capacities for clinical research studies, 2) developing a multi-center retrospective case series and prospective study to characterize the disease course, prognostic factors, and outcomes with current treatment approaches, 3) exploring local processes for convalescent plasma collection from MERS-CoV-infected patients (directed donations) and using it as a therapeutic intervention in the context of a

				prospective observational study with serial virologic sampling, and 4) developing a randomized controlled trial of an antiviral intervention.
	WHO Consultative meeting to determine a public health research agenda on MERS-CoV	Cairo, Egypt	15–16 Dec 2013	Discuss and agree on the urgent need for conducting a multinational case–control study to better understand and identify risk factors and types of exposure that result in infection; a seroepidemiological investigation to evaluate the extent of infection in contacts, risk factors for infection and detect sub-clinical human infection; and further animal studies to identify the animal reservoir of the virus, including the intermediate host, if any. Determine an implementation plan and identify roles and responsibilities for conducting the agreed research studies on MERS-CoV. Review the current public health recommendations in view of the recent findings and develop a set of rapid recommendations as part of an interim guidance on appropriate measures for prevention of risk of infection among high risk groups.
	WHO Consultative meeting to determine a public health research agenda on MERS-CoV	Riyadh, Saudi Arabia	2–3 Mar 2014	Finalize a protocol for a case-control study to determine significant exposure factors for MERS-CoV infection. After the meeting, the protocol was finalized and distributed to the affected countries.
	Emergency Committee Meetings under the International Health Regulations (IHR)	Teleconference	9 Jul 2013; 17 Jul 2013; 25 Sep 2013; 4 Dec 2013; 14 May 2014; 17 Jun 2014	The Emergency Committee providing expert technical advice to the WHO Director-General in accordance to the IHR (2005). The Committee constantly concluded that based on available information, and using a risk-assessment approach, the conditions for a PHEIC have not been met.
	2nd scientific advisory board meeting of the WHO collaborating center for mass gathering medicine	Riyadh, Saudi Arabia	28-29 Apr 2014	The 2nd Scientific Advisory Board Meeting of the Global Center for Mass Gathering Medicine, Ministry of Health, Riyadh, Kingdom of Saudi Arabia, met to discuss risk of infectious diseases and research and surveillance during Hajj. Due to the on-going outbreak of MERS-CoV and especially the recent increase in case detection in Jeddah, the agenda for the second day was focused on MERS-CoV, both in relation to the risk it presents for the forthcoming Umrah during Ramadan and the Hajj, but also in the Kingdom of Saudi Arabia and the Middle East in general. The Ministry of Health used the opportunity to ask the Scientific Advisory Board to review the MERS-CoV situation globally with specific attention to MERS in the country and review case definition, infection control guidelines and risk assessment to nationals, residents, health care workers, family contacts, camel owners, and travelers to KSA, and the future control.
	World Health Assembly	Geneva, Switzerland	May 2014	Following discussions held between WHO and SAU, during the last World Health Assembly in May 2014, WHO and SAU strengthened collaboration for international response to MERS-CoV in SAU under the International Health Regulations (2005). After these discussions, a WHO team was deployed to SAU from 21 May to 30 June 2014. WHO has offered further support to the Ministry of Health and the Command and Control Centre (CCC) in Jeddah in coordination of the international response to the outbreak.
	Regional technical consultation meeting, convened by FAO and the Ministry of Agriculture and Fisheries of the Sultanate of Oman	Muscat, Oman	20-21 May 2014	Declaration warned countries in the region and beyond of the need for public health and veterinary authorities to carry out coordinated investigations, and share information and results.Participants in the MERS consultation agreed that there is also a vital need to raise awareness among the public at large about the importance of seeking medical attention, the nature of the disease, and ways to avoid it.
	EMRO sub-regional workshop on improving infection prevention and control measures in health facilities for acute respiratory infection and specifically for MERS-CoV	Riyadh, Saudi Arabia	1-3 Jun 2014	IPC professionals from KSA, Kuwait, Lebanon, Egypt, Oman, Tunisia, Libya, Morocco, Bahrain, Jordan and Pakistan attended this workshop. At the end of the workshop, a framework was developed for improving health facilities preparedness for MERS focussing on strengthening IPC measures in healthcare settings.
II EPIDEMIC PHASE	Meeting of the OIE ad hoc Group on MERS-CoV Infection in Animals	Paris, France	15-17 Jul 2014	The group reviewed the WHO current interim general recommendations on MERS-CoV transmission from animals to humans and the interim recommendations for at risk groups. The group was supportive of the recommendations and suggested they should apply to countries where there was a risk of transmission of MERS-CoV from camels to humans. The group reviewed the current state of knowledge and made a series of recommendations with regard to animal health management, need for further studies, surveillance activities and on revising the OIE Q&A. The OIE Q&A have been amended accordingly.

	EMRO Workshop	Casablanca, Morocco	3-5 Sep 2014	The workshop focused on plans to enhance surveillance for severe acute respiratory infections for early detection, recognition and response to MERS-CoV, as well as public health measures that could effectively early detect any imported case of Ebola virus disease, as well as limit local transmission once a case associated with international travel is imported.
	WHO organized a meeting with medical missions of the top 10 countries sending pilgrims to Mecca for the Hajj	Jeddah, Saudi Arabia	16-17 Sep 2014	The participants reviewed and discussed overall preparedness measures for MERS-CoV during and after Hajj and identified better ways to enhance surveillance and exchange of information. A WHO team was deployed to Jeddah and Mecca during the Hajj to oversee the preparedness and surveillance activities related to MERS-CoV. This team provided support for coordination and information sharing between WHO, the SAU Ministry of Health and other countries sending large numbers of pilgrims.
	Emergency Committee Meetings under the International Health Regulations (IHR)	Teleconference	1 Oct 2014; 5 Feb 2015; 17 Jun 2015; 3 Sep 2015	The Emergency Committee, which comprises international experts from all WHO Regions, will provide expert technical advice to the WHO Director-General in accordance to the IHR (2005). The Committee constantly concluded that based on available information, and using a risk-assessment approach, the conditions for a PHEIC have not been met.
	FAO's and Qatar Supreme Counsil of Health Technical meetings on MERS-CoV	Doha, Qatar	27-29 Apr 2015	During the Doha and Cairo meetings, participants shared published and unpublished research findings; discussed the urgent need to modify control measures and to develop evidence-based measures and risk communication materials based on the accumulation of evidence linking camels to human infection; discussed the enhancement of joint collaboration between human and animal health sectors in field investigations, surveillance and research; and agreed on regular meetings to discuss and disseminate information to affected countries and the international community. The Qatar meeting concluded with the “Doha Declaration”, which calls for more joint animal/human investigations of cases and recommended that all animals that test PCR positive for MERS-CoV, regardless of an epidemiologic link to humans, be reported immediately to OIE and to national health authorities.
TRANSITION PHASE	The 13th Conference of the World Organisation for Animal Health’s (OIE) Regional Commission for the Middle East	Kaslik, Lebanon	10-14 Nov 2015	Several key issues involving the Member Countries of the region, with special emphasis on the following matters were also deliberated including camel disease control in the Middle East, including an update on MERS-CoV situation.
	WHO consultation of leading experts to develop a roadmap for MERS-CoV activities as part of the blueprint agenda and Workshop on prioritization of pathogens	Geneva, Switzerland	08–11 Dec 2015	At the meeting, four strategic goals were agreed upon in principle. The first is to establish a surveillance network of coronavirus laboratories as an early warning system to identify circulating species and strains in animal populations, new outbreaks in human populations and emerging strains in all populations. The second is to acquire a better understanding of MERS-CoV pathogenesis, natural history and veterinary and human epidemiology. The third is to develop, manufacture, test, license and use improved diagnostics, preventives and therapeutics that enable the interruption of transmission between humans and from dromedary camels to humans. The fourth is for the global donor community to establish a mechanism that provides a line of-sight for manufacturers from preclinical proof-of-concept studies to post-licensing procurement of MERS-CoV products, by initiating a public-health financial model for emerging pathogens prioritized by the WHO blueprint process.
	FAO Technical Meeting on Understanding MERS-CoV at the animal-human interface	Rome, Italy	21-22 Jan 2016	The goal of the meeting was to determine the current status of scienti c knowledge on MERS-CoV and identify major gaps that require further studies, in order to better understand the disease dynamics at the interface between humans and animals, and to develop practical approaches to control and minimize the impact of this virus. The meeting also aimed at fostering collaboration and partnerships between institutions and organizations working on MERS-CoV at the human-animal interface.
	EMRO training workshop aimed at initiating the sentinel site surveillance system for Severe Acute Respiratory Illness (SARI) and Influenza-Like Illness (ILI)	Riyadh, Saudi Arabia	15 -19 Jan 2017	It included collecting and handling the respiratory specimens, laboratory testing and reporting of respiratory illnesses, including MERS-CoV.

	WHO and The University of Hong Kong co-hosted an informal meeting to bring together public health and academic professionals	Hong Kong, China	13-14 Mar 2017	To discuss previously conducted studies of respiratory virus persistence and plan for future observational and experimental studies of environmental and air sampling of MERS-CoV. The participants in this meeting discussed the potential role of environmental contamination and airborne transmission of MERS-CoV in health-care settings based on their own research and developed a plan for future research to be conducted experimentally and observationally where MERS patients are treated.
ENZOOTIC PHASE	WHO EMRO hosted a consultation on emerging pathogens for laboratories (EDPLN) within the WHO Eastern Mediterranean Region.	Cairo, Egipty	22-23 Aug 2017	The participants in this meeting discussed laboratory capacity in the region and defined key priorities for laboratory activities on emerging pathogens, including MERS-CoV.
	FAO-OIE-WHO Global Technical Meeting	Geneva, Switzerland	25–27 Sep 2017	The meeting reviewed the latest scientific findings and identified and prioritized the global activities necessary to prevent, manage and control the disease. Critical needs for research and technical guidance identified during the meeting have been used to update the WHO R&D MERS-CoV Roadmap for diagnostics, therapeutics and vaccines and a broader public health research agenda.
	WHO EMRO scenario-based training	Beirut, Lebanon	25-28 Sep 2017	For Rapid Response Teams (RRT) in the region. The training covered early detection, field investigation and rapid response to outbreaks caused by novel respiratory pathogens, pandemic influenza and MERS-CoV.
	WHO EMRO conducted a training workshop for public health staff of the Ministry of Health of Lebanon	Lebanon	1-3 Oct 2017	Support on outbreak preparedness, detection, field investigation, development of a national RRT and emergency coordination during epidemics.
	14th Conference of the OIE Regional Commission for the Middle East	Istanbul, Turkey	2-6 Oct 2017	In addition, several additional key issues involving the Member Countries of the region, with special emphasis on the following matters were fruitfully deliberated including A fully functional regional network of expertise for camel diseases, CAMENET, undertaking research in camel disease diagnostics and epidemiology, including related to MERS-CoV
	WHO officials met with the Ministry of Health of Saudi Arabia	Saudi Arabia	18-21 Nov 2017	Senior officials responsible for MERS-CoV surveillance, case management, infection prevention and control, data collection and analysis, data base management and training met in Saudi Arabia. The Ministry of Health also arranged for a joint informal meeting with Ministry of Agriculture officials to discuss their ongoing surveillance and research activities in dromedary camel population. The objectives of the meetings were to outline WHO support for national activities on MERS-CoV in Saudi Arabia.
	WHO and FAO held a coordination meeting	Rome, Italy	27 Nov 2017	To plan coordinated MERS related activities at the animal human interface for 2017-2018.
	Ministry of Health of Saudi Arabia held a workshop	Saudi Arabia	30 Jan-3 Feb 2018	To update their National Guidance Update on MERS, in which WHO participated.
	WHO conducted their annual review of the WHO R&D Blueprint list of priority of diseases		6-7 Feb 2018	For the purposes of the R&D Blueprint, WHO uses a special tool for determining which diseases and pathogens to prioritize for research and development in public health emergency contexts. This tool seeks to identify those diseases that pose a public health risk because of their epidemic potential and for which there are no, or insufficient, countermeasures. The diseases identified through this process are the focus of the work of R& D Blueprint. This is not an exhaustive list, nor does it indicate the most likely causes of the next epidemic. Experts consider that given their potential to cause a public health emergency and the absence of efficacious drugs and/or vaccines, there is an urgent need for accelerated research and development for*: Crimean-Congo haemorrhagic fever (CCHF), Ebola virus disease and Marburg virus disease, Lassa fever, Middle East respiratory syndrome coronavirus (MERS-CoV) and Severe Acute Respiratory Syndrome (SARS), Nipah and henipaviral diseases, Rift Valley fever (RVF), Zika, and "Disease X". Disease X represents the knowledge that a serious international epidemic could be caused by a pathogen currently unknown to cause human disease, and so the R&D Blueprint explicitly seeks to enable cross-cutting R&D preparedness that is also relevant for an unknown "Disease X" as far as possible.
	WHO HQ and AFRO held a planning meeting		15-16 Feb 2018	To discuss MERS-CoV and emerging respiratory disease-related activities for the African Region.

	24th Tripartite Annual Executive Meeting	Paris, France	21-22 Feb 2018	Progress and plans for joint work on issues such as preparedness and response to emerging, re-emerging and neglected infectious diseases, antimicrobial resistance, and food safety were discussed.
	WHO and IVI held a joint symposium for MERS-CoV Vaccine Development	Seoul, Korea	26-27 Jun 2018	The meeting brought together more than 120 experts and professionals from industry, academia, international agencies and government. Progress on the development and trials of dromedary and human vaccines were presented.
	WHO EMRO training	Riyadh, Saudi Arabia	1-5 Jul 2018	Training on building national and subnational rapid response capacity for detection and response to MERS suspect cases and outbreaks.

Table S1.3. Description of guidelines published by international organizations (of WHO, FAO and OIE) throughout the epidemic phases addressing the response interventions targeted at MERS-CoV, April 2012 – December 2018 (n=16 guidelines).

Guideline	*Date*	*Content*
WHO's interim case definition of MERS for reporting to WHO	25 Sep 2012 updated 3 Jul 2013; 14 Jul 2014 and 14 Jul 2015; 26 July 2017	These case definitions have been revised based on new information collected since the previous definitions were published. WHO will continue to review and update them as new information becomes available. Note that these definitions are for classification and reporting. As such, they should not be taken as recommendations for when and whom to test. Surveillance recommendations and guidance on the investigation of cases of human infection with MERS-CoV can be found on the WHO coronavirus website.
WHO's interim recommendations for laboratory testing for MERS-CoV	Oct 2012, revised Dec 2012 updated Feb 2013, 16 Sep 2013; Sep 2014; 30 January 2018	The purpose of this document is to provide interim recommendations to laboratories and stakeholders involved in the laboratory testing for novel coronavirus. The recommendations have been prepared by WHO and reviewed by laboratory experts, including those with experience handling this virus and other coronaviruses, and also those with expertise in the development of diagnostic assays for coronaviruses. Part of this review process included a global conference call in late November 2012. WHO is closely monitoring developments related to this virus and will revise these recommendations when necessary. Unless revisions are made, this document will expire on 31 March 2013.
WHO's interim surveillance recommendations for human infection with MERS-CoV	Nov 2012 updated 27 Jun 2013, 14 Jul 2014, Jun 2018	This document summarizes WHO recommendations, and is not a comprehensive summary of current case reports. It is important to note that these WHO recommendations need to be implemented in different countries with varying resources and epidemiological patterns.
Interim guidance on Clinical management of severe acute respiratory infection when MERS-CoV infection is suspected (asymptomatic persons who are RT-PCR positive for MERS-CoV)	Mar 2013 updated 27 Jul 2015; 3 January 2018	The first edition of this document was published in 2013 and was revised in 2015. The current version is aligned with other WHO documents on case definitions and laboratory testing for MERS and incorporates changes to recommendations in supportive care of patients with critical illness based on evidence published since the last update. This document is intended for clinicians taking care of hospitalised adult and paediatric patients with severe acute respiratory infection (SARI) when MERS-CoV infection is suspected. It is not meant to replace clinical judgment or specialist consultation but rather to strengthen clinical management of these patients and provide references to up-to-date guidance.

WHO's Interim guidance on infection prevention and control during health care for probable or confirmed cases of MERS-CoV infection (WHO/MERS/IPC/15.1)	6 May 2013 updated 4 Jun 2015	This guidance reflects current understanding of MERS-CoV related to Infection Prevention and Control (IPC) and uses revised case definitions. The guidance is intended for health-care workers (HCWs), health-care managers, and IPC teams.
WHO's Interim guidance on Considerations for mass gathering events and MERS-CoV (WHO/MERS/MG/15.1 Rev.1)	3 Jun 2013 updated 5 Aug 2015; May 2018	This document presents organizers and hosts of international mass gatherings with proposed steps to improve public health preparedness to prevent, contain, and mitigate the impact of MERS-CoV. This document proposes elements of planning to be considered to prevent, contain and mitigate MERS-CoV infections during mass gathering events in the following areas: 1) Enhanced surveillance; 2) Command and control arrangements that link actions across agencies; 3) Travel health planning; 4) Clinical management of confirmed MERS-CoV cases; 5) Infection prevention and control; and 6) Risk communication.
WHO guidelines for investigation of cases of human infection with MERS-CoV	5 Jul 2013 updated May 2018	This document provides a standardized approach for public health authorities and investigators at all levels to plan for and conduct investigations around confirmed and probable cases of MERS-CoV infection. It should be read in conjunction with other detailed guidance referenced throughout the text, such as current laboratory testing guidelines and study protocols. It will be updated as necessary to reflect increased understanding of MERS-CoV transmission and control.
WHO's Travel advice on MERS-CoV for pilgrimages	25 Jul 2013 updated 1 Jun 2017	Description of advised actions for effective communication of risk information; actions for countries to take in preparation for Umra and Hajj and afterwards; actions at borders and for conveyances.
Initial Interview Questionnaire of Cases	30 Jul 2013 updated 24 May 2017	This form is designed to gather initial information about the potential exposures of a suspected or confirmed case of MERS- CoV infection in the 14 days before symptom onset. The interview should be conducted as soon as possible once the patient is suspected of having MERS-CoV infection. If the patient is unable to personally answer questions because of death or severity of illness, a close relative or friend can answer the questions for him or her. This form should be modified according to local needs and experience.
Interim guidance on home care for patients with MERS-CoV infection presenting with mild symptoms and management of contacts (WHO/MERS/IPC/18.1)	8 Aug 2013 updated June 2018	These recommendations reflect current understanding of MERS-CoV infection related to infection prevention and control (IPC) and public health measures. Specific WHO guidance on clinical management, infection control in health care, laboratory diagnostics, and surveillance has already been published (2–9). This document is complementary to WHO interim guidance on the management of asymptomatic persons who are positive for MERS-CoV in reverse transcriptase polymerase chain reaction (RT-PCR) assays (2).
WHO protocol on Sero-epidemiological Investigation of Contacts of MERS-CoV	09 Nov 2013	This protocol describes a cohort study design for a comprehensive assessment of all contacts of confirmed and probable MERS-CoV cases, including household, familial, social, and occupational contacts, to evaluate the spectrum of illness and risk factors associated with infection, routes and risk of transmission.
WHO's MERS-CoV transmission from animals to humans, and interim recommendations for at-risk groups	13 Jun 2014 updated 26 January 2018	Preliminary results from an ongoing investigation in Qatar show that people working closely with camels (e.g. farm workers, slaughterhouse workers and veterinarians) may be at higher risk of MERS-CoV infection than people who do not have regular close contacts with camels. In Qatar and several other countries, animals, including goats, cows, sheep, water buffalo, swine and wild birds, have been tested for antibodies to MERS-CoV, with no positive results. The absence of antibodies in these animals indicates that the likelihood of other animals having a substantial role in transmission of MERS-CoV is very low. These studies provide evidence that camels are a likely primary source of the MERS-CoV that is infecting humans.
Middle East Respiratory Syndrome Coronavirus (MERS-cov) Case Definition for Reporting to OIE	May 2017	This case definition was updated again in 2019.
WHO Target Product Profiles for MERS-CoV Vaccines	May 2017	This document describes the preferred and minimally acceptable profiles for 3 vaccines: Dromedary camel vaccine – for prevention of transmission of MERS-CoV among camels and from camels to humans; Human vaccine – for long term protection of persons at high ongoing risk of MERS-CoV such as healthcare workers and those working with potentially infected animals; Human vaccine – for reactive use in outbreak settings with rapid onset of immunity. These Target Product Profiles (TPPs) were developed through a consultation process with key stakeholders in human and animal health, scientific, funding and manufacturing communities. It is intended

		that they will guide and prioritize the development of vaccines. As new scientific evidence is generated, these TPPs may require further review and revision.
Interim Case Summary Form for rapid reporting of probable and confirmed cases of MERS-CoV infection to WHO	24 May 2017	v2 has been updated taking into consideration knowledge of potential and suspected risk factors for infection and severe disease
FAO information sheet: Dromedary camels and MERS-CoV: filling knowledge gap	Jun 2017	This information sheet presents some key facts on an emerging threat to public health globally which can cause severe respiratory infections in humans: the Middle East Respiratory Syndrome Coronavirus (MERS-CoV). It also describes the One Health coordinating role of FAO in reducing the risk of MERS-CoV at the human-animal inter- face by working with global partners such as the World Organisation for Animal Health and the World Health Organization. In particular, FAO monitors the situation; analyses available data; provides technical assistance and guidance to countries to improve understanding of the disease situation and help filling existing gaps in epidemiological knowledge; supports national laboratories to develop technical capacities; and assists countries in developing communication strategies.
